# Improving geographically extensive acoustic survey designs for modeling species occurrence with imperfect detection and misidentification

**DOI:** 10.1002/ece3.4162

**Published:** 2018-05-20

**Authors:** Katharine M. Banner, Kathryn M. Irvine, Thomas J. Rodhouse, Wilson J. Wright, Rogelio M. Rodriguez, Andrea R. Litt

**Affiliations:** ^1^ Department of Ecology Montana State University Bozeman Montana USA; ^2^ U.S. Geological Survey Northern Rocky Mountain Science Center Bozeman Montana USA; ^3^ U.S. National Park Service Upper Columbia Basin Network Inventory and Monitoring Program Bend Oregon USA; ^4^ Department of Animal & Rangeland Sciences Courtesy Faculty Oregon State University Cascades Bend Oregon USA; ^5^ Human and Ecosystem Resiliency and Sustainability Lab Oregon State University‐Cascades Bend Oregon USA

**Keywords:** bats, false‐positive occupancy models, manual review, monitoring, passive animal detectors, survey design, vetting

## Abstract

Acoustic recording units (ARUs) enable geographically extensive surveys of sensitive and elusive species. However, a hidden cost of using ARU data for modeling species occupancy is that prohibitive amounts of human verification may be required to correct species identifications made from automated software. Bat acoustic studies exemplify this challenge because large volumes of echolocation calls could be recorded and automatically classified to species. The standard occupancy model requires aggregating verified recordings to construct confirmed detection/non‐detection datasets. The multistep data processing workflow is not necessarily transparent nor consistent among studies. We share a workflow diagramming strategy that could provide coherency among practitioners. A false‐positive occupancy model is explored that accounts for misclassification errors and enables potential reduction in the number of confirmed detections. Simulations informed by real data were used to evaluate how much confirmation effort could be reduced without sacrificing site occupancy and detection error estimator bias and precision. We found even under a 50% reduction in total confirmation effort, estimator properties were reasonable for our assumed survey design, species‐specific parameter values, and desired precision. For transferability, a fully documented r package, OCacoustic, for implementing a false‐positive occupancy model is provided. Practitioners can apply OCacoustic to optimize their own study design (required sample sizes, number of visits, and confirmation scenarios) for properly implementing a false‐positive occupancy model with bat or other wildlife acoustic data. Additionally, our work highlights the importance of clearly defining research objectives and data processing strategies at the outset to align the study design with desired statistical inferences.

## 1 INTRODUCTION

Remotely deploying acoustic recording units (ARUs) to survey cryptic animals is an important tool for ecology and conservation biology (e.g., Blumstein et al., 2011; Newson, Bas, Murray, & Gillings, 2017; Parsons & Szewczak, 2009). Acoustic recording units are capable of collecting detection/non‐detection data on focal species noninvasively and with minimal effort across broad geographic extents, making coordinated monitoring practical and feasible, even for sensitive species (e.g., Acevedo & Villanueva‐Rivera, 2006; Loeb et al., 2015). Despite these advantages, the sheer volume of data collected by ARUs often necessitates automated species identification via classification software, resulting in the potential for two types of detection errors: imperfect detection and misidentification. Imperfect detection occurs when the focal species is present but no calls are recorded, or calls are recorded but none are identified as the focal species. Misclassification errors result from the classification software incorrectly assigning at least one recorded call to the focal species, when in fact, the species is absent from a site. Statistical analyses of ARU datasets can provide decision‐makers with critical baseline information about the probability of species occurrence (site occupancy) and species distributions for at‐risk species (e.g., McClintock, Bailey, Pollock, & Simons, 2010; Rodhouse et al., 2015). Standard occupancy models can be used to address these research goals while accounting for imperfect detection, but the modeling framework assumes no misidentification errors (MacKenzie et al., 2006). When misidentification errors are ignored, estimators from standard occupancy models can be biased (e.g., McClintock et al., 2010; Miller et al., 2011; Royle & Link, 2006) and lead to unreliable management and conservation decisions.

Bat acoustic surveys that set out ultrasonic microphones for recording bat echolocation calls provide acute examples of how misidentification and imperfect detection may arise and complicate statistical inferences. Challenges associated with traditional capture and visual methods coupled with the increased risk of multiple threats (e.g., Hammerson, Kling, Harkness, Ormes, & Young, 2017; Jones, Jacobs, Kunz, Willig, & Racey, 2009; O'Shea, Cryan, Hayman, Plowright, & Streicker, 2016) have accelerated the widespread use of ARUs for surveying bats. Broad‐scale monitoring programs have been initiated across Europe, North America, and elsewhere (e.g., Barlow et al., 2015; Jones et al., 2013; Loeb et al., 2015; Roche et al., 2011; Walters et al., 2012) and rely in part on coordinated acoustic surveys. The complication is that shared echolocation call characteristics from morphologically and ecologically similar bat species can result in incorrect species assignments from automated identification software and misidentification errors for the focal species (Russo, Ancillotto, & Jones, 2017; Russo & Voigt, 2016; Rydell, Nyman, Eklof, Jones, & Russo, 2017). Imperfect detection can occur when all echolocation calls from the focal species are of such low quality that they are filtered out during call processing. Another source is when all calls from the focal species do not exhibit enough distinguishing characteristics to receive a single‐species classification resulting in no species assignment or a group classification such as low‐ or high‐frequency bat. Additionally, the focal species could be present despite having none of its calls recorded.

Detection error rates arising from automated identification software are impacted by the focal species' call characteristics and behavior, the choice of detector (e.g., type and model), detector settings (e.g., gain level), detector placement in relation to environmental clutter (e.g., vegetation that may alter call behavior), the classification software used, and the call processing workflow employed. To promote coherency between these important considerations and the eventual modeling framework used, we diagram a generalized workflow for recording, processing, and verifying bat echolocation call files in Section 2.2. This workflow diagramming strategy highlights how typical practices may influence the appropriateness of certain modeling approaches for bat acoustic data and serves as a conceptual model for practitioners interested in designing ARU‐based surveys for any taxa.

Standard occupancy models can account for imperfect detection if multiple within‐season visits are made to each site (e.g., MacKenzie et al., 2002, 2006), a sampling design commonly used for bat acoustic surveys (e.g., Gorrensen, Miles, Todd, Bonaccorso, & Weller, 2008; Rodhouse et al., 2015; Weller, 2008). Furthermore, automated species identifications can be manually verified by a human to remove misidentification errors and provide confirmed detections prior to analysis (e.g., Wright, Irvine, & Rodhouse, 2016). For species that can be verified consistently and truly, the amount of effort and expertise required for this approach is impractical for large‐scale coordinated monitoring. The difficulty posed by this verification burden can lead to the naive modeling approach of applying a standard occupancy model to unverified bat acoustic data, effectively ignoring misidentification errors. We propose an alternative option of explicitly modeling misidentifications in a false‐positive occupancy model.

Three classes of false‐positive occupancy models are outlined in Chambert, Miller, and Nichols (2015): site confirmation models (Miller et al., 2011, 2013), calibration models (Chambert et al., 2015; Ruiz‐Gutierrez, Hooten, & Grant, 2016), and the observation confirmation (OC) model (Chambert et al., 2015). Chambert, Waddle, Miller, Walls, and Nichols (2018) recently introduced another type of OC model that has the potential to extend inferences to include estimates of relative abundance of some taxa. False‐positive occupancy models require auxiliary information about true site occupancy from a subset of sites or calibration information about the detection device's misidentification rate to ensure estimates of detection probabilities are unique. To our knowledge, Clement, Rodhouse, Ormsbee, Szewczak, and Nichols (2014) provide the only application of a false‐positive occupancy model to a bat acoustic survey. Clement et al. (2014) drew on mist‐netted bats as true detections from a subset of sites to inform the probability of misidentification using a multiple method site confirmation model (Miller et al., 2011). However, capturing bats is invasive, costly, and quickly becomes impractical for geographically extensive surveys. It is also debatable whether hand captures constitute true detections for certain bat species, as many species are morphologically cryptic (e.g., Rodhouse, Scott, Ormsbee, & Zinck, 2008; Rodriguez & Ammerman, 2004; Weller, Scott, Rodhouse, Ormsbee, & Zinck, 2007). Bat acoustic data pose challenges for the calibration model as well, as the libraries of echolocation calls used to build classification software for automated species identifications are not always made under realistic conditions (Russo et al., 2017). The OC models, on the other hand, show promise for leveraging information from bat acoustic surveys while potentially reducing the manual verification burden.

We extend Chambert et al.'s (2015) OC model to accommodate known sources of heterogeneity in occupancy and detection probabilities and allow for spatially explicit estimates of occurrence and detection probabilities. Otherwise, ignoring potential sources of heterogeneity in detection probabilities could result in biased estimators (Miller et al., 2015). Further, our OC model extension allows for more flexibility in allocating confirmation effort than is afforded by the original formulation of the OC model in Chambert et al. (2015). We focus on whether using our extended OC model for analyses can provide a way to increase efficiency of ARU‐based surveys through reduced confirmation effort. Our investigation into confirmation effort is complementary to the one presented in Chambert et al. (2018). Here, we focus on exploring a simpler OC model that does not rely on specifying an appropriate statistical distribution for nightly bat activity.

We use simulation to compare the three approaches for addressing misidentification errors in statistical modeling of ARU‐based surveys: (a) *REMOVE*, removing them and applying a standard occupancy model; (b) *IGNORE*, ignoring them and applying a standard occupancy model; and (c) *MODEL*, using our extended OC model to explicitly account for them in the modeling framework. We compare approaches with respect to their estimator properties such as bias, precision, and coverage. We also explore how the allocation of confirmation effort affects OC parameter estimator properties. Our simulations were based on species‐specific parameter estimates from real bat acoustic data and illustrate the importance of using available pilot data when considering sample size questions. Importantly, we provide a fully documented R (R Core Team, 2016) package, OCacoustic, for conducting customized investigations. To improve access and applicability for practitioners, all of our functions incorporate the common r‐formula syntax used in glm and occu. The package is bundled with an extended vignette providing instructions and guidelines for its use (Appendix S3).

## 2 MATERIALS AND METHODS

### 2.1 General terminology

The unit of analysis or sample unit for occupancy models is commonly a predefined spatial unit (MacKenzie et al., 2006). In our application, sites are defined as 10‐km × 10‐km grid cells within the state of Oregon, USA, Rodhouse et al. (2012), where the area was chosen based on focal species behavior and analysis objectives. In general, we suggest a probabilistic sampling design for choosing sites (e.g., a design based on the generalized randomized‐tessellation stratified (GRTS) algorithm; Stevens & Olsen, 2004). Observations arising from different sites are assumed to be independent, as are those arising from different visits to the same site. We define a single visit as a one‐night deployment of an ARU to a unique location within a site. The replication needed to account for imperfect detection could be spatial replicates with multiple ARUs deployed within a site (if a spatial unit) on the same night, or temporal replicates with one ARU deployed for multiple nights at the same location within a site (although see Wright et al. (2016) for potential drawbacks). It is assumed that the occupancy status of a site is the same for all visits. The standard occupancy model and the OC model both use this terminology and require these assumptions.

**Figure 1:**
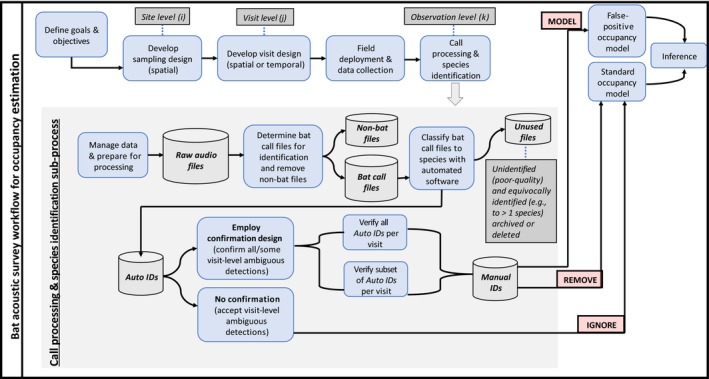
A bat acoustic survey workflow diagram. This workflow begins with goals and objectives (occupancy modeling in this example) and ends with inferences and conclusions. All intermediate steps influence downstream tasks (blue boxes). A critical step in any analysis is outlining the workflow to facilitate conversation among collaborators and ensure consistency among data collection, analysis, and dissemination of results *prior to deploying ARUs*. The focus of this diagram is occupancy modeling using bat acoustic data, but similar workflow diagrams can be created for different analysis objectives and/or different animals of interest (e.g., insects, frogs, and birds). The style of this diagram was inspired by the business workflow modeling software, Bizagi Modeler (http://www.bizagi.com)

Many bat echolocation call files can be recorded during a visit and detection/non‐detection could be considered at two different levels: the observation level (i.e., individual recordings of echolocation calls) or the visit level (i.e., aggregating individual recordings up to a visit). For both levels, we define two types of detections: *ambiguous detections* which can include misidentifications of the focal species, and *unambiguous detections* without misidentification errors. Species identifications made by automatic software constitute ambiguous detections, whereas, those that are a posteriori verified by a qualified expert are unambiguous. We define *verification* as the process for obtaining unambiguous observation‐level detections, *confirmation* as that for unambiguous visit‐level detections, and the *confirmation design* as the visit‐level detections that are chosen to be confirmed. Although the confirmation of visit‐level ambiguous detections is done through verification at the observation level, the verification strategy employed will impact modeling options. Therefore, an important step in the planning phase of any ARU‐based survey is diagramming the acoustic data workflow (e.g., Figure 1).

### 2.2 Bat acoustic data workflow

Diagramming the conceptual workflow for any study begins with clearly articulated inferential goals and objectives (start of Figure 1). Here, our objective is to use information from bat acoustic surveys to estimate site occupancy probabilities for one focal species with uncertainty. Most importantly, the focal species must be detectable acoustically and the number of sites, number of visits, and visit design should be based on species‐specific behavioral characteristics (e.g., MacKenzie et al., 2006). Before deployment, detector locations and settings should be chosen specifically for species of interest following a consistent protocol (e.g., Loeb et al., 2015; NPS, 2016).

After calls have been recorded, workflow decisions during “Call Processing and Species Identification” (Figure 1) directly affect the final set of echolocation calls used in statistical analysis, and in turn, inferences from the three modeling approaches we consider. Call processing begins by removing *Non‐Bat Files* (e.g., wind and insects) from the collection of *Raw Audio Files*, and retaining only *Bat Call Files*. Then, using classification software (e.g., Sonobat, Kaleidoscope, etc.), *Bat Call Files* are assigned to one of three types of classifications: single‐species identification (*Auto IDs*, Figure 1); combination of potential species or frequency group identification (e.g., EPFU/LANO or HiF; *Unused Files*, Figure 1); or no ID (*Unused Files*, Figure 1). *Auto IDs* assigned to the focal species constitute observation‐level ambiguous detections, and they are used to determine visit‐level ambiguous detections: ambiguously detected if at least one *Auto ID* is identified to the focal species, undetected otherwise. Some *Raw Audio Files* ending up in the *Non‐Bat Files* or *Unused Files* data‐storage containers could be missed calls from the focal species. The estimated imperfect detection rate encapsulates all possible ways a focal species may end up with zero *Auto IDs* including the possibility of the species not being recorded at all.

Next, a decision about the confirmation design must be made: employ a confirmation design and have an expert verify *Auto IDs*, or accept visit‐level ambiguous detections and proceed with the *IGNORE* approach by naively fitting a standard occupancy model. If a confirmation design is chosen, it is assumed that calls from the focal species can be verified consistently and truly by an expert. Verified *Auto IDs* receive corresponding *Manual IDs*, which are then used to confirm visit‐level ambiguous detections: unambiguously detected (confirmed) if at least one *Manual ID* is from the focal species, undetected otherwise. The *REMOVE* approach requires confirmation for all visit‐level ambiguous detections, but it does not require all *Auto IDs* within visits to be verified. Conversely, for the *MODEL* approach, the OC model does not require confirmation for all visits but does assume that all (or a representative sample from all) of the *Auto IDs* within visits are verified. We discuss implications for commonly employed verification strategies used for our *REMOVE* approach and suggest modifications to ensure consistency with OC model assumptions in Section 4.

### 2.3 The OC model applied to bat acoustic data

**Figure 2:**
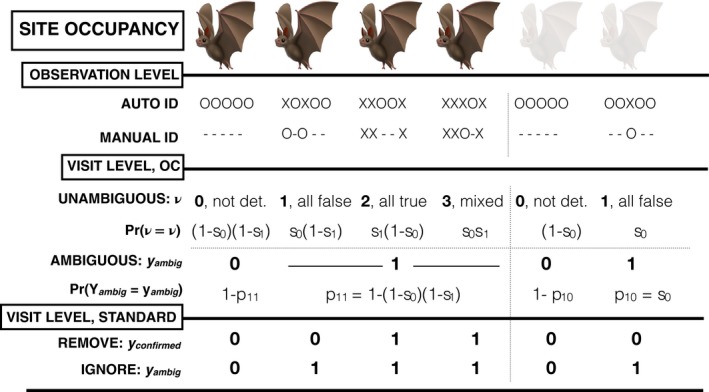
Diagrammatic representation of the OC model applied to bat acoustic data. True site occupancy status is represented by the color of the bat (dark = occupied, light = unoccupied). At the observation level, *Auto IDs* are single‐species classifications receiving an ID to the focal species (X) or to a different species (O). For the OC model, visit‐level ambiguous detections (*Y*_ambig_) are determined by the aggregation of *Auto IDs*, and unambiguous detections (*ν*) by the (dis)agreement between all of the *Auto IDs* and the *Manual IDs* collected during the visit. Probabilities for each visit‐level OC model outcome are provided below the outcomes, where *s*_1_[*s*_0_] = the probability that during a single visit, at least one observation (*Auto ID*) is recorded and then correctly[incorrectly] identified to the focal species by the classification software. Detection/non‐detection data for *REMOVE* (*Y*_confirmed_) and *IGNORE* (*Y*_ambig_) are obtained through the aggregation of *Manual IDs* and *Auto IDs* to the visit level, respectively

In the context of bat acoustic surveys, the OC model (Chambert et al., 2015; pg. 336) uses observation‐level *Auto IDs* and *Manual IDs* to estimate visit‐level detection and misclassification probabilities (concept diagram in Figure 2) conditional on the ARU workflow employed (e.g., Figure 1). This model assumes the occupancy status of the *i*th site (*i* = 1, 2, ···, *n*) is a Bernoulli random variable with a constant probability of ψ (*Z*_*i*_ ∼ Bernoulli(*ψ*): *Z*_*i*_ = 1 if the focal species occupies the *i*th site, *Z*_*i*_ = 0 otherwise). It also assumes species detections during visits occur at occupied sites (visit‐level true detections) with probability *p*_11_ and at unoccupied sites (visit‐level misidentifications) with probability *p*_10_. That is, visit‐level ambiguous detections during the *j*th (*j* = 1, 2, ···, *J*_*i*_) visit to the *i*th site also arise from a Bernoulli distribution, $Y_{{\mathrm{ambig}}_{\mathrm{ij}}}∼\text{Bernoulli}(p_{11}\times Z_{i}+p_{10}\times \left(1-Z_{i}\right))$, where $y_{{\mathrm{ambig}}_{\mathrm{ij}}}=1$ if at least one *Auto ID* is identified to the focal species during visit *j* to site *i*, $y_{{\mathrm{ambig}}_{\mathrm{ij}}}=0$ otherwise (*y*_ambig_ in Figure 2).

The visit‐level unambiguous detection (*ν* in Figure 2) is assumed to be a multinomial random variable with levels defined by the (dis)agreement between the *observation‐level Auto IDs* and their corresponding verified *Manual IDs*: (*ν* = 0) “no detections”‐ no *Auto IDs* identified to the focal species implying no detection to confirm (Figure 2, Columns 1, 5), (*ν* = 1) “all false” – all *Auto IDs* identified to the focal species are overturned and their corresponding *Manual IDs* denote a different species or “no ID” (Figure 2, Columns 2, 6), (*ν* = 2) “all true” – all *Auto IDs* identified to the focal species are verified by *Manual IDs* that denote the focal species (Figure 2, Column 3), and (*ν* = 3) “mixed” – at least one *Auto ID* identified to the focal species is overturned and at least one is verified (Figure 2, Column 4). The OC model assumes that at least one *Auto ID* is recorded and subsequently correctly identified to the focal species by the classification software with probability *s*_1_ (at least one observation‐level true‐positive) and incorrectly identified to the focal species with probability *s*_0_ (at least one observation‐level misidentification). Thus, conditional on the occupancy status of the site, the probability space for the visit‐level unambiguous detection during the *j*th visit to the *i*th site (*ν*_*ij*_) is defined as,

(1) $$\text{Pr}\left(\nu_{\mathrm{ij}}=\left\{0,1,2,3\right\}|Z_{i}=1\right)=\left\{\left(1-s_{0}\right)\left(1-s_{1}\right),\;s_{0}\left(1-s_{1}\right),\;s_{1}\left(1-s_{0}\right),\;s_{0}s_{1}\right\}$$

(2) $$\text{Pr}\left(\nu_{\mathrm{ij}}=\left\{0,1,2,3\right\}|Z_{i}=0\right)=\left\{s_{0},1-s_{0},0,0\right\}.$$

The detection probabilities for the visit‐level ambiguous detections can also be written in terms of *s*_0_/*s*_1_: Pr(*Y*_*ij*_ = 1|*Z*_*i*_ = 1) = *p*_11_ = *s*_1_ + *s*_0_ − *s*_1_ *s*_0_, and Pr(*Y*_*ij*_ = 1|*Z*_*i*_ = 0) = *p*_10_ = *s*_0_, fully parameterizing the OC model by two detection probabilities and the probability of occurrence: *s*_1_, *s*_1_, and *ψ*, respectively (see also, Figure 2).

We provide more flexibility in how much verification effort is allocated to each site by allowing for a combination of ambiguous (*y*_ambig_) and unambiguous (*ν*) detections within a site. The likelihood for our extended OC model is written as,

(3) $$\begin{array}{cc} L( & \psi_{i},s_{0_{\mathrm{ij}}},s_{1_{\mathrm{ij}}}\left|I{\left(C\right)}_{\mathrm{ij}},\nu_{\mathrm{ij}},y_{{\mathrm{ambig}}_{\mathrm{ij}}}\right)=\\ & \prod_{i=1}^{n}\left[\psi_{i}\times \left(\prod_{j=1}^{J}I{\left(C\right)}_{\mathrm{ij}}\left(\text{Pr}\left(\nu_{\mathrm{ij}}|z_{i}=1\right)\right)+\left(1-I{\left(C\right)}_{\mathrm{ij}}\right)p_{11_{\mathrm{ij}}}^{y_{{\mathrm{ambig}}_{\mathrm{ij}}}}{\left(1-p_{11_{\mathrm{ij}}}\right)}^{1-y_{{\mathrm{ambig}}_{\mathrm{ij}}}}\right.\right)\\ & \left.+\left(1-\psi_{i}\right)\times \left(\prod_{j=1}^{J}I{\left(C\right)}_{\mathrm{ij}}\left(\text{Pr}\left(\nu_{\mathrm{ij}}|z_{i}=0\right)\right)+\left(1-I{\left(C\right)}_{\mathrm{ij}}\right)p_{10_{\mathrm{ij}}}^{y_{{\mathrm{ambig}}_{\mathrm{ij}}}}{\left(1-p_{10_{\mathrm{ij}}}\right)}^{1-y_{{\mathrm{ambig}}_{\mathrm{ij}}}}\right)\right], \end{array}$$

where *I*(*C*)_*ij*_ = 1 if the *j*th visit to the *i*th site is confirmed and *I*(*C*)_*ij*_ = 0 otherwise. Relationships between site‐level covariates (**X**_*i*_) and site occupancy (*ψ*_*i*_) and site‐ and visit‐level covariates ($W_{l_{\mathrm{ij}}}$) and detection probabilities ($s_{l_{\mathrm{ij}}}$; for l = 0,1) are modeled using the logit link function as, $\text{logit}\left(\psi_{i}\right)=\theta X_{i}$ and $\text{logit}\left(s_{l_{\mathrm{ij}}}\right)=\beta_{l}W_{l_{\mathrm{ij}}}$, for *l *=* *0,1, respectively. The occupancy and detection coefficients are represented by the θ and $\beta_{l}$ vectors, respectively.

One source of heterogeneity in the observation process for bat acoustic data is the overall quality of calls obtained during a visit. We expect overall call quality to be a function of detector and microphone placement, the amount of “environmental clutter” (e.g., vegetation, rocks, and water surfaces) near the detector, weather (e.g., wind and rain), and potentially other sources. A reasonable proxy to use for call quality is the total number of *Auto IDs* recorded during a visit, as only calls of a certain quality will ultimately receive single‐species *Auto IDs*. We let *K*_*ij*_ be the total number of *Auto IDs* recorded by the detector deployed at visit *j* in site *i*, and we note that *K*_*ij*_ is always greater than or equal to zero. We expect that at some value of *K*_*ij*_, the proxy for quality ceases to substantially influence detection probabilities (i.e., the difference between observing 1 and 50 *Auto IDs* is more reflective of a change in call quality between visits than is the difference between observing 1,001 and 1,050 *Auto IDs*). Therefore, for our application and simulations, we assume a logit‐linear relationship between the natural log of *K*_*ij*_ + 1 (adding one to ensure the value being logged is greater than zero) and the detection probabilities,

(4) $$\text{logit}\left(s_{l_{\mathrm{ij}}}\right)=\beta_{0,s_{l}}+\beta_{1,s_{l}}\left(\log\left(K_{\mathrm{ij}}+1\right)\right);\;l=0,1.$$

If available, visit‐level covariates explaining heterogeneity in detection probabilities (e.g., software packages, regional classifiers, and “clutter”) could be directly incorporated into the mean structure through Equation (4). Similarly, covariates also could be included at the site level (e.g. habitat type and average elevation) to account for heterogeneity in site occupancy using a logit link function on *ψ*_*i*_.

### 2.4 Simulation study design

We were interested in whether the extended OC model was a viable alternative to the *REMOVE* approach. We conducted a simulation study comparing estimation of occupancy (*ψ*) among *REMOVE*,* IGNORE*, and our extended OC model with a confirmation design where all visits within all sites were confirmed (i.e., all unambiguous data). We included the *IGNORE* approach to corroborate that ignoring misidentifications from bat species classification software in a standard occupancy model can result in erroneous conclusions (e.g., Clement et al., 2014). To explore the impacts of the confirmation design on parameter estimation using our extended OC model, we also investigated nine different confirmation designs: all, half, or a quarter of sites with all, half, or a quarter of the visits being confirmed (i.e., contributing information in terms of *ν* (unambiguous) rather than *y*_ambig_ (ambiguous) in Figure 2). We explored all possible combinations for a range of parameter values ($\psi ,\left(\beta_{0,s_{1}},\beta_{1,s_{1}}\right),\;\;\text{and}\;\;\left(\beta_{0,s_{0}},\beta_{1,s_{0}}\right)$), chosen to represent realistic species‐specific characteristics based on empirical data (see Appendix S1 for empirical results). For this study, we specified *ψ* to reflect narrowly and widely distributed species (Label: low (L), high (H); “Occupancy” in Table 1). We set the regression coefficients associated with correct automated identifications (at least one call recorded and correctly classified to the focal species during a visit $\left(\beta_{0,s_{1}},\beta_{1,s_{1}}\right)$) to represent species that were hard, average, or easy to detect (Label: low (L), medium (M), high (H), “Baseline detect” in Table 1). Similarly, we set regression coefficients associated with automated misidentifications $\left(\beta_{0,s_{0}},\beta_{1,s_{0}}\right)$ to represent species that were more easily or less easily confused with other species by the classification software (i.e., harder to misidentify or easier to misidentify; labeled low (L) or high (H) for “Baseline misID” in Table 1, respectively).

**Table 1:** Parameter combinations representing species characteristics based on empirical data (see Appendix S1). Three‐letter labels reflect levels for *ψ*, $\left(\beta_{0,s_{1}},\beta_{1,s_{1}}\right)$, and $\left(\beta_{0,s_{0}},\beta_{1,s_{0}}\right)$, respectively. For example, HMH represents a widely distributed species (*ψ* = 0.8) with average detection ($\left(\beta_{0,s_{1}},\beta_{1,s_{1}}\right)$ = (0, 1.6)) that can be easy to misidentify (i.e., easily confused with other species) in the automated identification classification process ($\left(\beta_{0,s_{0}},\beta_{1,s_{0}}\right)$ = (−2.2, 1.5))

Label	Occupancy (*ψ*)	Baseline detect ($\beta_{0,s_{1}}$)	Baseline misID ($\beta_{0,s_{0}}$)
HHL	Widely distributed	Easier	Harder
HLL	Widely distributed	Harder	Harder
LLL	Narrowly distributed	Harder	Harder
LLH	Narrowly distributed	Harder	Easier
HMH	Widely distributed	Average	Easier

Label	$\{\psi ,\left(\beta_{0,s_{1}},\beta_{1,s_{1}}\right),\left(\beta_{0,s_{0}},\beta_{1,s_{0}}\right\}$
HHL	{0.8, (1.5, 1.6), (−3, 1.5)}
HLL	{0.8, (−1.4, 1.6), (−3, 1.5)}
LLL	{0.2, (−1.4, 1.6), (−3, 1.5)}
LLH	{0.2, (−1.4, 1.6), (−2.2, 1.5)}
HMH	{0.8, (0, 1.6), (−2.2, 1.5)}

For each parameter combination, we generated 500 realizations of data (datasets) assuming the same sampling design as that used in our empirical data (*n *=* *84 sites, and *J*_*i*_ = 4 visits for all *i*), and assuming unambiguous visit‐level observations arose from the data‐generating process described by the extended OC model. That is, we assumed a confirmation design where all visits within all sites were confirmed resulting in unambiguous data (*ν*‐values) for all visits. We first generated true occupancy states (*Z*_*i*_) from a Bernoulli(*ψ*) distribution. Then, we obtained realistic covariate values for each visit by sampling with replacement from *K*‐values in the empirical data. Using *Z*_*i*_ and the *K*_*ij*_‐values, we produced visit‐level detection probabilities ($s_{0_{\mathrm{ij}}}/s_{1_{\mathrm{ij}}}$) following Equation (4) and generated the unambiguous dataset by taking random draws from the appropriate multinomial distribution for each site (*ν*_*ij*_|*Z*_*i*_ = 1 or *ν*_*ij*_|*Z*_*i*_ = 0, Equations (1) and (2)). Using the unambiguous dataset, we obtained a dataset for the *REMOVE* approach (*y*_confirmed_‐values) and a dataset for the *IGNORE* approach (*y*_ambig_‐values) according to the relationships conveyed in Figure 2 (e.g., $\nu_{11}=1\Rightarrow y_{{\mathrm{ambig}}_{11}}=1$, $y_{{\mathrm{confirmed}}_{11}}=0$ for site 1 and visit 1).

We then investigate reduced confirmation efforts for the *MODEL* approach. We let *C*_*p*,*d*_ denote a confirmation design where *p* indicates the proportion of confirmed sites (*p *=* *1, 0.5, 0.25), and *d* indicates the number of confirmed visits within those sites (*d *=* *1, 2, 4). To construct a dataset for the confirmation design *C*_*p*,*d*_, we randomly selected *p* × 84 sites to receive *d* confirmed visits. We remained consistent with the extended OC model by assigning confirmation to visits with ambiguous detections $\left(y_{{\mathrm{ambig}}_{\mathrm{ij}}}=1\right)$ prior to those with non‐detections $\left(y_{{\mathrm{ambig}}_{\mathrm{ij}}}=0\right)$. The dataset was completed using unambiguous data (*ν*_*ij*_‐values) for visits with *I*(*C*_*p*,*d*_)_*ij*_ = 1 and ambiguous data ($y_{{\mathrm{ambig}}_{\mathrm{ij}}}$‐values) for visits with *I*(*C*_*p*,*d*_)_*ij*_ = 0. Our approach to data generation used the unambiguous dataset to obtain datasets for the *REMOVE* approach, *IGNORE* approach, and the eight other confirmation designs for the OC model, effectively minimizing Monte Carlo (MC) error in our simulation investigations. Thus, we minimized MC error in our comparisons of modeling approaches and confirmation designs.

All programming was performed in r Statistical Software version 3.3.2 (R Core Team, 2016). Maximum‐likelihood (ML) estimates for OC model parameters were found by minimizing the negative log‐likelihood (negative log of Equation (3)) using nlm, and corresponding standard errors (*SE*s) were calculated by inverting the Hessian. The occu function from the unmarked package (Fiske & Chandler, 2011) was used to fit the two standard occupancy models (*REMOVE* and *IGNORE*). For each approach and each dataset, we saved parameter estimates, their corresponding approximate (±2 *SE*) 95% confidence intervals, and whether or not the data‐generating parameter values were captured in their corresponding intervals. We also tracked three types of computational errors: if the negative log‐likelihood function could not be evaluated at randomly generated starting values, if the minimization failed to converge, and if there was an error in inverting the Hessian. We defined *convergence* as a dataset that resulted in an ML‐estimation process where the Hessian was invertible and parameter estimates and their corresponding SEs were reasonable (estimate ≤10 and CI width ≤30 for parameters on the logit scale, CI width ≤0.7 for parameters on the probability scale). For all realizations of data that converged, we computed the average point estimate (average of individual estimates), average approximate 95% confidence interval (lower/upper endpoint defined by the average of the individual lower/upper estimates), and coverage for each parameter.

## 3 SIMULATION STUDY RESULTS

For brevity and clarity in Sections 3.1–3.3, we focus on results from the five parameter combinations in Table 1 and a subset of confirmation designs. General patterns for other parameter combinations and confirmation designs were similar, and results are included in Appendix S2.

### 3.1 Directly comparing *IGNORE*,* REMOVE*, and *MODEL*

**Figure 3:**
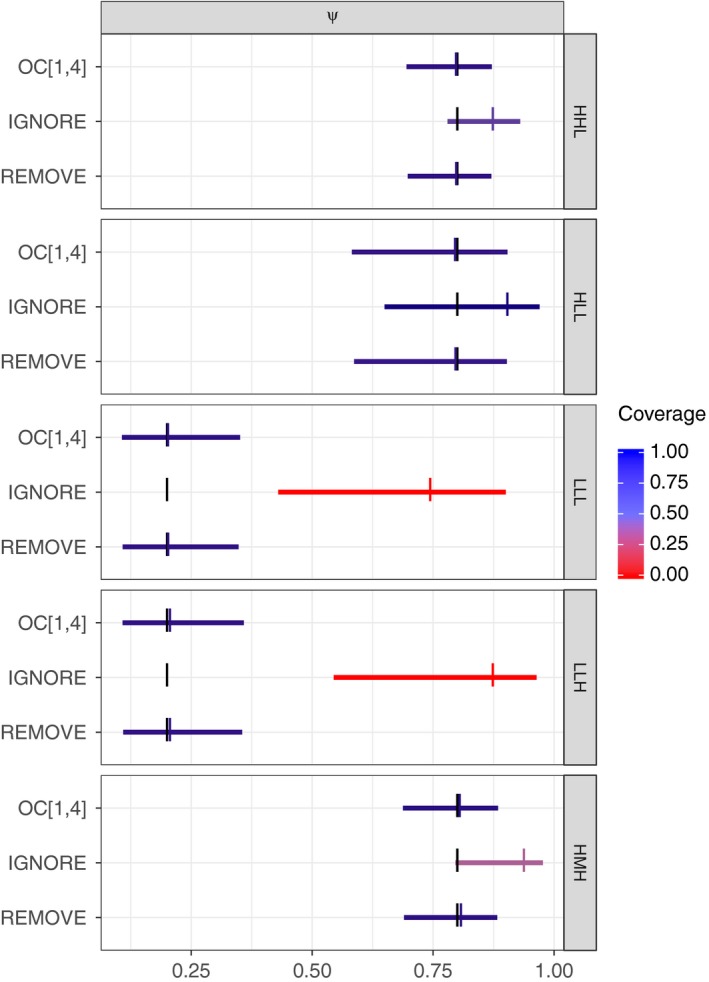
Average approximate 95% confidence intervals for occupancy (*ψ*) using the *REMOVE* approach (bottom CI), the *IGNORE* approach (middle CI), and the OC model fit to only unambiguous data (top CI). We generated five hundred simulated datasets for each parameter combination (rows). Three‐letter labels for parameter combinations indicate assumed occupancy (L = narrowly distributed, H = widely distributed), baseline detection (L = hard to detect, M = average, H = easy to detect), and baseline misidentification (L = hard to misidentify, H = easy to misidentify). Assumed parameter values are shown with large black vertical tick marks, and their corresponding average estimates are shown with colored tick marks. Coverage is indicated by color (0 = red, 1 = blue). Simulation created using oc_sim_gen, and Figure created using FNOC_psi_compare in OCacoustic

The average 95% CIs for occupancy probability were nearly identical for the *REMOVE* approach and the *MODEL* approach where the OC model was applied to all unambiguous data (Figure 3, top and bottom CIs). This suggests the two approaches produce similar inferences regarding species occupancy for the species‐specific characteristics we considered. Furthermore, both approaches resulted in reasonably unbiased estimators with nominal coverage (≥0.95) and enough precision to be informative (e.g., all rows, Figure 3). The *IGNORE* approach (middle CIs, Figure 3), on the other hand, resulted in consistently positively biased estimators for species‐specific characteristics considered, often accompanied by poor coverage probabilities (all but HLL, Figure 3).

For all three approaches, precision, coverage, and bias of the occupancy estimator varied based on the species‐specific characteristics assumed during data generation. In particular, precision was sensitive to detectability. For example, we observed wider confidence intervals for occupancy in widely distributed species that were also assumed to be hard to detect (HLL in Figure 3) than we did when their detection was assumed to be average or easy (HMH and HHL in Figure 3; see also, Figures S2.1 and S2.2). This pattern held for *IGNORE*,* REMOVE*, and OC (all three CIs in Figure 3). Interestingly, there was little to no difference in the precision, coverage, and bias of the occupancy estimator when comparing high versus low misidentification rates for species with the same assumed occupancy and baseline detectability (LLH vs. LLL in Figure 3, see also Figures S2.1 vs. S2.2).

### 3.2 Comparing confirmation design scenarios within the MODEL approach

**Figure 4:**
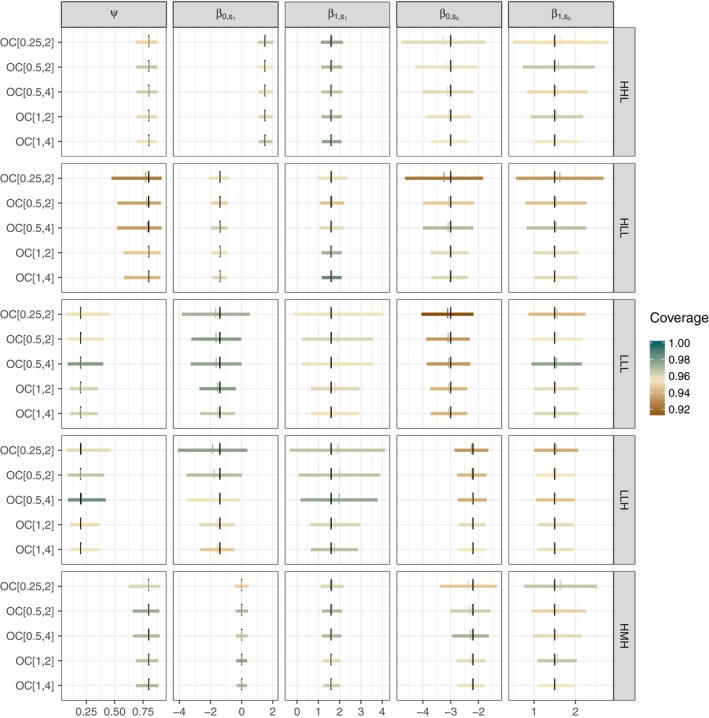
Average approximate 95% confidence intervals for each OC model parameter (column), computed from 500 simulated datasets generated assuming five parameter combinations (rows) for the OC model, assuming five confirmation designs (denoted *C*_*p*,*d*_ on the *y*‐axis, where *p* indicates the proportion of confirmed sites and *d* indicates the number of confirmed visits within those sites). Three‐letter row‐labels indicate assumed occupancy (L = narrowly distributed, H = widely distributed), baseline detection (L = hard to detect, M = average, H = easy to detect), and baseline misidentification (L = hard to misidentify, H = easy to misidentify). Assumed parameter combinations are shown with large black vertical tick marks along with corresponding average estimates in colored tick marks. Coverage is indicated by color, *note that the scale for coverage ranges from 0.9 (brown) to 1 (green), rather than 0 to 1* Figure created using oconly_compare_gen in OCacoustic

Fitting the OC model to all unambiguous data (OC_[1,4]_ in Figure 4) provides a reference case producing similar results to the verification intensive *REMOVE* approach. Here, we compare different confirmation designs to assess the OC model's potential to reduce verification effort. We observed that for a fixed number of confirmed visits (e.g., compare CIs for OC_[1,2]_, OC_[0.5,2]_, OC_[0.25,2]_, Figure 4), the precision for all OC model parameters decreased (CIs became wider) as the proportion of confirmed sites decreased; this observation held for all parameter combinations (see also, Figures S2.1 and S2.2). Whereas, for a given proportion of confirmed sites, decreasing the number of confirmed visits resulted in little difference in the uncertainty of estimated OC model parameters (e.g., compare CI widths OC_[1,4]_ to OC_[1,2]_ and OC_[0.5,4]_ to OC_[0.5,2]_, Figure 4; see also Figures S2.1 and S2.2). Although this pattern held for all confirmation designs, many realized datasets generated assuming a confirmation design with only one unambiguous visit did not converge. This suggests that to successfully fit the extended OC model to species with characteristics like those that we assumed, the confirmation design should have at least two confirmed visits within confirmed sites (see Section 3.3).

Therefore, we found the properties of the extended OC model estimator depended on the confirmation design and the species‐specific characteristics assumed during data generation. For harder‐to‐detect species, we observed less than nominal coverage probabilities (<0.95) for all parameters when only two visits were confirmed within a quarter of the sites. For narrowly distributed species, we observed estimator bias for the partial regression coefficients associated with true detection. The widely distributed species, on the other hand, showed bias for partial regression coefficients associated with misidentification. This bias was most pronounced for confirmation designs with half or a quarter of the sites confirmed (e.g., rows HLL, LLL, LLH, and top three CIs). Similarly, for a given occupancy and misidentification rate, we observed wider average CIs for occupancy with harder‐to‐detect species and less confirmed data (HHL vs. HLL, all CIs; Figure 4). Interestingly, however, the baseline misidentification rate (when moving from L to H, or 0.05 to 0.10 on the probability scale) did not largely affect the confirmation design required to produce an unbiased and precise extended OC model estimator (LLL vs. LLH in Figure 4).

### 3.3 Computational limitations

**Figure 5:**
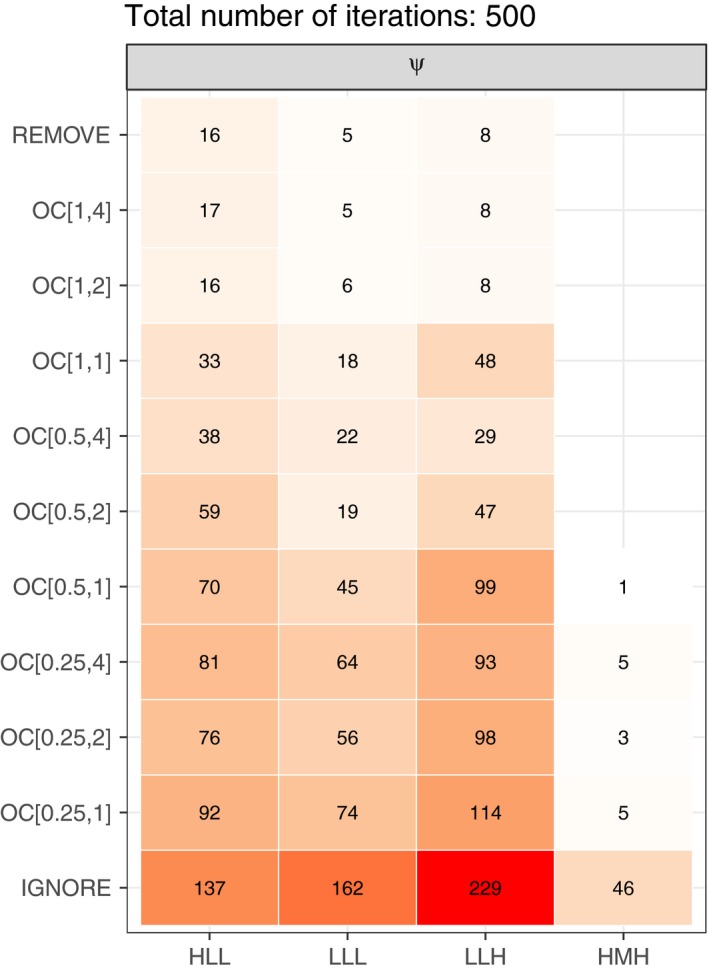
Heatmap for the occupancy parameter, *ψ*, showing the number of iterations out of 500 that the optimizer failed to converge for a given method (*y*‐axis) and assumed parameter combination (*x*‐axis). Three‐letter row‐labels indicate assumed occupancy (L = narrowly distributed, H = widely distributed), baseline detection (L = hard to detect, M = average, H = easy to detect), and baseline misidentification (L = hard to misidentify, H = easy to misidentify). The confirmation effort denoted *C*_*p*,*d*_ on the *y*‐axis, where *p* indicates the proportion of confirmed sites and *d* indicates the number of confirmed visits within those sites. Color intensity displays the number of iterations that resulted in the optimizer failing to converge. *Note that for a widespread species that was easy to detect and hard to misidentify (HHL), there were no convergence issues for any of the approaches*. Figure created using converge_plot in OCacoustic

For confirmation designs with unambiguous data in only a quarter of the sites or only one visit within sites, a large number of realizations failed to converge (Figure 5, see also Figure S2.3). Similarly, the *IGNORE* approach displayed convergence issues more frequently than the *REMOVE* approach or any of the confirmation designs with the extended OC model. Fitting the extended OC model to datasets generated assuming easier‐to‐detect species (label = − M − or − H −) and confirmation designs with more confirmed visits (OC_[1,4]_ OC_[1,2]_) resulted in minimal convergence issues for all parameters (Figure 5, see also Figure S2.3). This pattern suggests there is a minimum confirmation effort required for fitting the extended OC model and the minimum will vary based on the assumed species characteristics.

## 4 DISCUSSION

We outline an occupancy modeling framework that accounts for misidentification errors and simultaneously reduces the manual verification burden required for defensible inferences to inform conservation and management. This framework also allows for variability in detection probabilities related to field deployment conditions and the classification software used for automated identification; making it a flexible approach for geographically extensive surveys. Standard occupancy models require all visit‐level misidentification errors to be eliminated from a dataset (i.e., *REMOVE* approach). In comparison, our *MODEL* approach accounts for possible misidentification errors while still allowing for some ambiguous detections to be included in the analysis. In this way, the extended OC model inherently provides increased efficiency of ARU‐based surveys by maintaining tolerable levels of uncertainty with reduced confirmation effort. Generally, we found confirming more sites resulted in more precise extended OC model estimators compared with confirming more visits within sites, but the “optimal” confirmation design was dependent on assumed species characteristics (detectability and occupancy).

We suggest diagramming the workflow (Section 2.2) as a first step in the survey design process because it highlights key decisions that may impact statistical inferences. Here, we focus on how the selection of *Auto IDs* for verification impacts the proper interpretation of parameter estimates. During visits, large volumes of *Auto IDs* can be recorded, making verifying all observation‐level ambiguous detections impractical. Therefore, in practice, the following verification strategy is often employed for confirming visits: sort *Auto IDs* based on some a priori measure of call quality (e.g., probability calculated based on an artificial neural network, number of call pulses within a file classified to a species) and verify the “top” calls until enough have been verified that the verifier feels confident the species was present during the visit (e.g., a criterion of 3–5 calls)—or—until all *Auto IDs* have been overturned (i.e., correcting a visit‐level misidentification). This type of strategy is typically employed to increase efficiency with the *REMOVE* approach, but it is problematic for the OC model. Interpretations of detection probabilities estimated by the OC model become conditional on the specific subset of *Auto IDs* used for verification and the criterion chosen for confirmation. Because of this, it is essential that methods for call processing and verification accompany results from statistical analyses. Importantly, to enable broader and more defensible syntheses among projects and years, we recommend a verification strategy where all, or a random sample of all *Auto IDs*, are reviewed so that results are compatible and comparable (random sample also suggested in Chambert et al., 2018).

For simplicity, we presented the verification process for bat acoustic data in the context of a single focal species. In reality, verification is done for *all* potential species expected at each visit location. That is, the observation has multiple outcomes, with each outcome representing a potential species available for detection. For example, non‐detections for focal species A are, in fact, ambiguous detections for species B (i.e., *AutoID* = “species B”, a different species). Therefore, detection information about species B is available in the analysis for focal species A, but is ignored by the extended OC model. Further extensions to accommodate multiple species (e.g., community occupancy models) could exploit this valuable information and are worthy of investigation.

Our extended OC model incorporates observation‐level information into the analysis through the agreement/disagreement of ambiguous and unambiguous detections of the same observation, but the analysis is aggregated to the visit level. Recently, Chambert et al. (2018) model the count of ambiguous detections using a zero‐truncated Poisson distribution, and they discuss linking occupancy and relative abundance. However, equating nightly bat activity (number of *Auto IDs* recorded by a stationary detector) as a measure of relative abundance is more controversial for bats compared with anurans or birds (Hayes, 1997). Guillera‐Arroita, Lahoz‐Monfort, van Rooyen, Weeks, and Tingley (2017) also directly incorporate information at the observation level into their model, but the number of *Auto IDs* obtained during a visit can be so large that they cannot be handled by the Binomial distributions underlying their model. Essentially, by aggregating to the visit level, we avoid the need for additional model complexity to account for likely overdispersion in nightly bat activity. However, more methodology that directly uses observation‐level information and also accounts for the idiosyncrasies (e.g., volume, data processing pipeline, ad correlation among recorded calls) of acoustic data could be the focus of future work.

Consistent with other work (e.g., Clement et al., 2014; Newson et al., 2017), we found evidence of species misidentification errors from classification software and, if ignored, occupancy was severely overestimated, supporting the need for verification. We assumed the *Manual IDs* were consistent and true, but if acoustic data are verified by more than one expert, this assumption becomes more tenuous, such that standardizing the workflow is key. Currently, human verification provides the most reliable source of unambiguous detections for bat acoustic data, but this is subject to change. If call libraries for classification software used to obtain automatic species identifications improve and become more representative of conditions observed in the field, the calibration model (see Chambert et al., 2015; Ruiz‐Gutierrez et al., 2016) has the potential to eliminate verification from the acoustic data workflow entirely, effectively removing all costs associated with a manual confirmation design. As discussed in Russo and Voigt (2016), quality calibration information is not currently found among the published literature. Until then, we advocate using the extended OC model to reduce costs associated with the verification process for bat acoustic data, when appropriate. The OC model rests on the assumption that verification is consistent and true. This appears to be a reasonable assumption for most bats species that occur within a given faunal assemblage (see Fritsch & Bruckner, 2014; Russo & Voigt, 2016; Rydell et al., 2017), but becomes tenuous for rare species, species that are difficult to manually verify, or when multiple verifiers with different levels of experience are working on the same region of interest (Fritsch & Bruckner, 2014; Rydell et al., 2017). For rare bat species or less experienced verifiers, an entirely different survey design and analysis strategy should be pursued (e.g., using captured bats as in Clement et al., 2014) and the assumptions of that approach must also be carefully assessed.

Diagramming the workflow associated with wildlife acoustic data can help facilitate coherency among acoustic call processing and species identification decisions and subsequent statistical analysis and interpretations. In addition to providing functions like those available in unmarked (e.g., occu) for fitting the extended OC model, our r package (OCacoustic) provides the capacity for researchers to conduct their own investigations into design requirements (e.g., sample size and a desired level of uncertainty) prior to collecting data. OCacoustic facilitates exploring trade‐offs in extended OC model estimator precision and bias related to number of sites, number of visits, covariate structures at the site level and visit level, assumed data‐generating parameter values (ideally coming from estimates from pilot data), and confirmation designs. Importantly, OCacoustic provides a design tool to increase efficiency of future animal surveys that rely on ARUs to collect detection/non‐detection data for estimating spatially explicit occurrence probabilities (species distribution maps) to inform conservation and management.

## CONFLICT OF INTEREST

None declared.

## AUTHOR CONTRIBUTION

K. Banner, K. Irvine, T. Rodhouse, W. Wright, and A. Litt conceived the ideas and were involved in discussions regarding statistical methodology. T. Rodhouse and R. Rodriguez led the development of the conceptual workflow model, and all other authors contributed. R. Rodriguez wrote critical sections of Supporting S1 and manually reviewed the bat acoustic data. K. Banner created the R package and vignette. K. Banner led the writing of the manuscript with significant support from K. Irvine, A. Litt, and T. Rodhouse. All authors contributed critically to the drafts and gave final approval for publication.

## DATA ACCESSIBILITY

Our data are included in our R package which is archived in Katharine Banner's USGS BitBucket repository (https://my.usgs.gov/bitbucket/users/kbanner_usgs.gov/repos/ip-092225/browse/DataS1).
