# Supplementary material for: Improving geographically extensive acoustic survey designs for modeling species occurrence with imperfect detection and misidentification
Source: Ecol Evol. 2018 May 20;8(12):6144–56. doi: 10.1002/ece3.4162 (PMC6024138; doi:10.1002/ece3.4162)
Supplement: Supplementary file 1 [file ECE3-8-6144-s001.pdf]

Supporting S1: Application to bat acoustic data
Improving geographically extensive acoustic survey designs for modeling species occurrence with imperfect detection and misidentification

Katharine M. Banner [*,†] Kathryn M. Irvine [‡] Thomas J. Rodhouse [§,¶]
Wilson J. Wright [*] Rogelio M. Rodriguez [||] Andrea R. Litt [*]

April 13, 2018

S1 Application to empirical data

We applied the extended OC model to bat acoustic data to obtain realistic parameter estimates for our simulation study. These data are a collection of echolocation call files recorded by D500X ultrasonic detectors (Pettersson Elektronik, AB, Uppsala, Sweden) across the state of Oregon between June 6 and September 19, 2016. Sites were defined by and selected from an areal $10km \times 10km$ grid-based sample frame covering the contiguous United States (Ch. 3.2 in Loeb et al., 2015). Eighty-four sites were sampled and seventy-nine were subsequently processed with the common inferential objective of obtaining detection/non-detection data for all bat species expected in the region (i.e., for occupancy models); we used these seventy-nine sites in our analyses. The sampling design loosely followed recommendations provided by the North American Bat Monitoring Program (NABat; see Ch. 3 in Loeb et al., 2015) with a few modifications. According to the NABat program, sites are selected based on a probabilistic, spatially-balanced approach (i.e., Generalized Random-Tessellation Stratified; Stevens and Olsen 2004) which composed ∼60% of the sites surveyed during our study. Another ∼40% of sites surveyed were selected from sites that had the greatest number of visits during the legacy Bat Grid, a regional bat survey effort conducted by the U.S. Forest Service and Bureau of Land Management from 2003 - 2010 (Ormsbee et al., 2006). These legacy Bat Grid sites were selected for a separate analysis of comparison with historical data. Other

[*]Montana State University, Department of Ecology, Bozeman, MT 59717-3460, USA
[†]katharine.banner@montana.edu
[‡]U.S. Geological Survey, Northern Rocky Mountain Science Center, Bozeman, MT 59715, USA
[§]U.S. National Park Service, Upper Columbia Basin Network Inventory and Monitoring Program
[¶]Oregon State University Cascades, Courtesy Faculty, Department of Animal & Rangeland Sciences, Bend, Oregon 97702, USA
[||]Zotz Ecological Solutions, LLC, Bend, OR, 97709, USA

departures from the NABat protocol included an increased survey period into September (NABat: 1 June to 30 July) and limiting the number of nights surveyed to one night as suggested in Wright et al. (2016) (NABat: four nights).

Sites were partitioned into four quadrants (visits, assumed to produce independent observations) and the goal was to deploy detectors for one night within each quadrant. If this approach was not possible due to logistical constraints (e.g., land access), then multiple detectors were sometimes deployed within the same quadrant. In our analysis, we treat all audio files recorded by multiple detectors within the same quadrant as information for that quadrant only. Therefore, between one and four spatial visits were obtained for each site, and 227 total quadrants sampled. Within each quadrant, detectors were typically placed near suitable bat habitat, that is, landscape features amenable to drinking, foraging, and commuting. Microphones were elevated $\sim 3m$ off the ground and oriented horizontally within a distance of $\sim$2-5m towards the habitat feature of interest. Detectors were set to record at least 30 minutes prior to sunset to 30 minutes prior to sunrise. Detector calibration was not feasible as this was a large-scale project using many detectors from several equipment owners. Therefore, for consistency in detection quality and range among detectors, we used standard settings for all detectors: sampling frequency of 500 kHz, pre-trigger disabled (recommended for passive monitoring to ensure battery life throughout visits), recording length of 5 seconds, high pass filter disabled, auto-recording enabled, trigger sensitivity of medium (a signal will be recorded when approximately nine or less oscillations in the waveform exceeds the trigger level during approximately a 20 ms duration), input gain of 45, trigger level of 160 (implying detector triggers on approximately -16 dB relative to the maximum level of the signal) and interval of 0 (Reichert et al., 2017).

Raw Audio Files obtained during visits were automatically filtered to remove *Non-Bat Files* (see Figure 1) using a combination of SCAN'R Version 1.8.2 (Binary Acoustic Technology, Tucson, AZ, USA; `www.binaryacoustictech.com`) and Kaleidoscope Version 3.1.1 (Wildlife Acoustics, Concord, MA, USA; `www.wildlifeacoustics.com`). Filtering thresholds were set to include a wide range for individual call parameters (e.g., frequency and duration). The resulting *Bat Call Files* were auto-classified using the Pacific Northwest Classifier in Sonobat 4.0.7 (Arcata, CA, USA; `www.sonobat.com`) using the following settings: autofilter enabled, 5 kHz filter, classify enabled, write data enabled, append species codes to filenames enabled, acceptable call quality of 0.80, sequence decision threshold of 0.90, and max number of calls to consider per file of 16. Fourteen species that were detectable acoustically and were expected in the region were considered individually for site occupancy. For each species, manual verification was done for all visits that resulted in at least one *Auto ID* identified the species (i.e., for each visit-level ambiguous detection). The verification workflow employed was such that the highest quality *Auto IDs* were verified until the verifier was confident that the species was present during the visit resulting in a visit-level unambiguous detection - OR - until all *Auto IDs* were overturned, correcting a visit-level misidentification. The same expert, who is a bat biologist with 19 years of experience in recording and analysis of bat acoustic data, performed all of the manual verification. Therefore, it was reasonable to assume manual verification was consistent and true, resulting in unambiguous visit-level detection data for the fourteen species considered.

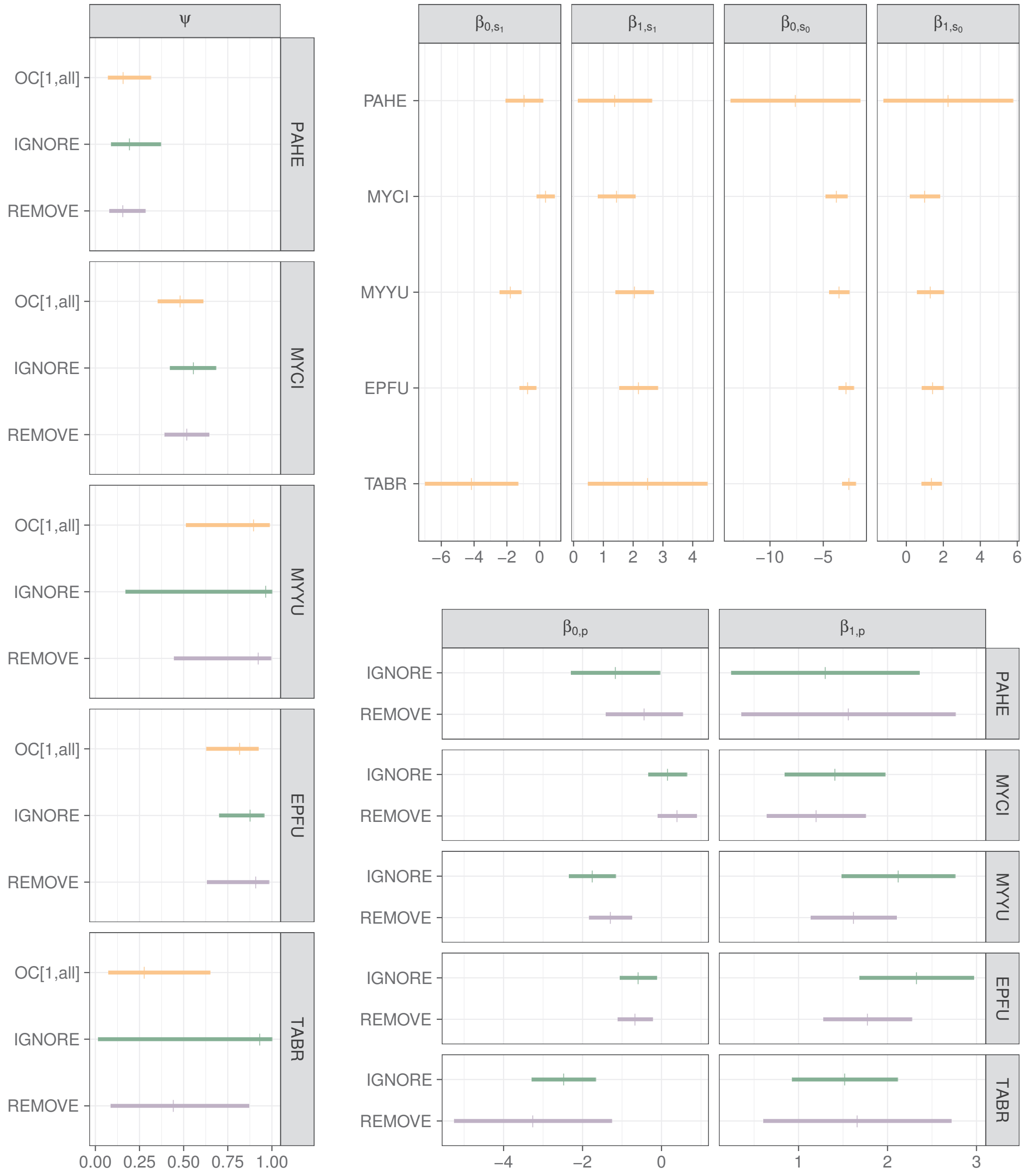

Figure S1.1: Estimates and approximate 95% ($\pm 2SE$) CIs for occupancy (ψ) for three modeling scenarios, $REMOVE$ (purple), $IGNORE$ (green), and $OC_{1,all}$ (gold) for each species (row) are shown in the leftmost plot. The top right plot shows approximate 95% CIs for the intercept and slope coefficients representing the linear relationship between $logit(s_1)$ and $log(K+1)$ (β_{0,s_1} & $\beta 1, s_1$), and the intercept and slope coefficients for the linear relationship between $logit(s_0)$ and $log(K+1)$ (β_{0,s_0} & $\beta 1, s_0$) from the $OC_{1,all}$ model. The bottom right plot shows the approximate 95% CIs for the intercept and slope coefficients representing the linear relationship between $logit(p)$ and $log(K+1)$ ($\beta_{0,p}, \beta_{1,p}$) for the two standard occupancy models ($REMOVE$ in purple and $IGNORE$ in green), where p is the probability of detecting a bat when it is there and the model assumes no misidentification errors.

We compared three common approaches: the $REMOVE$, the $IGNORE$, and our extended OC model applied to unambiguous data for all visits ($OC_{1,all}$). Here, we present

results for the five species that motivated the species-specific parameter combinations in our simulation study (canyon bat [*Parastrellus hesperus*: PAHE], western small-footed myotis [*Myotis ciliolabrum*: MYCI], Yuma myotis [*Myotis yumanensis*: MYYU], big brown bat [*Eptesicus fuscus*: EPFU], and Mexican free-tailed bat [*Tadarida brasiliensis*: TABR]). We used R Statistical Software Version 3.3.2 for all computations (R Core Team, 2016). The example dataset used here, our code for processing call file data, fitting the three approaches, and plotting results for comparisons is available in our R package (`OCacoustic`) on the author's USGS BitBucket page `https://my.usgs.gov/bitbucket/users/kbanner_usgs.gov/repos/ip-092225/browse/DataS1`. An extended vignette/tutorial for using our package is available in Supporting S3.

We analyzed these data solely for the purpose of obtaining realistic parameter combinations to use in our simulations, therefore, we do not make any species-specific inferences about bats in Oregon from these results. Rather, we focus on the range of estimated parameter values observed. We found evidence of false positives in automatic acoustic detections for most bat species (back-transformed CIs >> 0 on the probability scale), and we also observed a positive relationship between $log(K+1)$ and $logit(s_0)$ for four of the five species investigated (confidence intervals were strictly positive). We found slightly higher estimates for the occupancy parameter in the *IGNORE* approach than the *REMOVE* approach, and results from the OC model applied to unambiguous data were, in general, more consistent with those from the *REMOVE* than with those from the *IGNORE* model (e.g., MYCI, PAHE, TABR, Figure S1.1). This was most pronounced for narrowly distributed species and for those that were difficult to detect (e.g., PAHE and TABR in Figure S1.1). However, uncertainty around these estimates was largely due to the sparsity in the real data (15 sites with one visit, 14 with two visits, 16 with three visits, and 34 with four visits), indicating a need for more complete sampling or more data to obtain useful inferences from all of these approaches.

References

Loeb, S. C., Rodhouse, T. J., Ellison, L. E., Lausen, C. L., Reichard, J. D., Irvine, K. M., Ingersoll, T. E., Coleman, J. T. H., Thogmartin, W. E., Sauer, J. R., Francis, C. M., Bayless, M. L., Stanley, T. R., and Johnson, D. H. (2015). A plan for the north american bat monitoring program (nabat). Report SRS-208, United States Department of Agriculture.

Ormsbee, P., Zinck, J., Szewczak, J., Patrick, L., and Hart, A. (2006). Benefits of a standardized sampling frame: an update on the "bat grid.". *Bat Research News*, 47(4).

R Core Team (2016). *R: A Language and Environment for Statistical Computing*. R Foundation for Statistical Computing, Vienna, Austria.

Reichert, B., Lausen, C., Corben, C., Livengood, K., Szewczak, J., Weller, T., Loeb, S., Rodriguez, R., Britzke, E., Hohoff, T., Siemers, J., Burkholder, B., and Herzog, C. (2017). Nabat guide to acoustic detector settings. Technical report, North American Bat Monitoring Program.

Stevens, D. and Olsen, A. (2004). Spatially balanced sampling of natural resources. *Journal of the American Statistical Association*, 99:262–278.

Wright, W. J., Irvine, K. M., and Rodhouse, T. J. (2016). A goodness-of-fit test for occupancy models with correlated within-season revisits. *Ecology and Evolution*, 6(15):5404–5415.
