# Supplementary material for: Improving geographically extensive acoustic survey designs for modeling species occurrence with imperfect detection and misidentification
Source: Ecol Evol. 2018 May 20;8(12):6144–56. doi: 10.1002/ece3.4162 (PMC6024138; doi:10.1002/ece3.4162)
Supplement: Supplementary file 2 [file ECE3-8-6144-s002.pdf]

Supporting S2: Simulation study results
Improving geographically extensive acoustic survey designs for modeling species occurrence with imperfect detection and misidentification

Katharine M. Banner *† Kathryn M. Irvine ‡ Thomas J. Rodhouse § ¶
Wilson J. Wright * Rogelio M. Rodriguez ‖ Andrea R. Litt *

April 13, 2018

S2 Results

Here, we provide all of the results from our simulation investigation. We present results for assumed parameter combinations representing species that are harder for the classification software to misidentify (Low [L = 0.5 on prob scale] baseline misidentification rate, Figure S2) separately from those for assumed parameter combinations representing species that are easier for the software to misidentify (High [H = 0.1 on prob scale] baseline misidentification rate, Figure S3). We do this to show how little the baseline true detection or misidentification rate affected the trade-offs observed in confirmation design efforts with respect to average CI width, bias, and coverage. Slight improvements in properties of estimators were observed for assumed parameter combinations with harder-to-misidentify species as compared to those with easier-to-misidentify species. A vignette/tutorial for tailoring simulation studies to meet research needs using `OCacoustic` is provided as Supporting S3.

*Montana State University, Department of Ecology, Bozeman, MT 59717-3460, USA
†`katharine.banner@montana.edu`
‡U.S. Geological Survey, Northern Rocky Mountain Science Center, Bozeman, MT 59715, USA
§U.S. National Park Service, Upper Columbia Basin Network Inventory and Monitoring Program
¶Oregon State University Cascades, Courtesy Faculty, Department of Animal & Rangeland Sciences, Bend, Oregon 97702, USA
‖Zotz Ecological Solutions, LLC, Bend, OR, 97709, USA

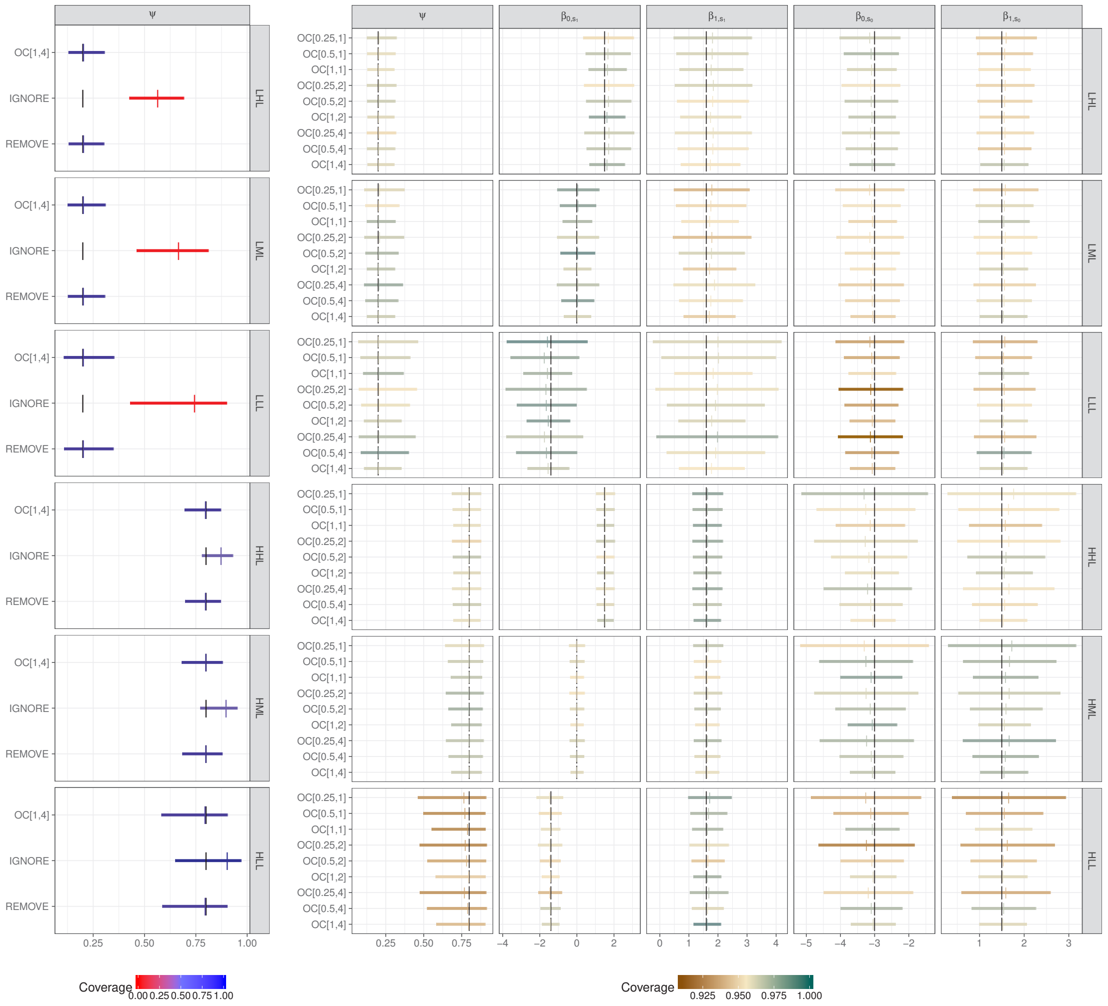

Figure S2.1: **Assumed data-generating parameter combinations for harder-to-misidentify species**: Left: Comparison of average approximate 95% CIs, bias (distance between assumed parameter combinations (shown as rows) used in data generation (black ticks) and average estimates (colored ticks), and coverage (0 = red, 1 = blue) for three approaches (*REMOVE*, *IGNORE*, and OC model fit to unambiguous data). Right: Comparison of the same results as those compared among the three models, but for the OC model applied to all confirmation designs. *Note that the coverage for the OC model confirmation design comparisons ranges from 0.9 (brown) to 1 (green).* Both: Three-letter row-labels indicate assumed occupancy (L = narrowly distributed, H = widely distributed), baseline detection (L = hard to detect, M = average, H = easy to detect), and baseline misidentification (L = hard to misidentify, H = easy to misidentify)

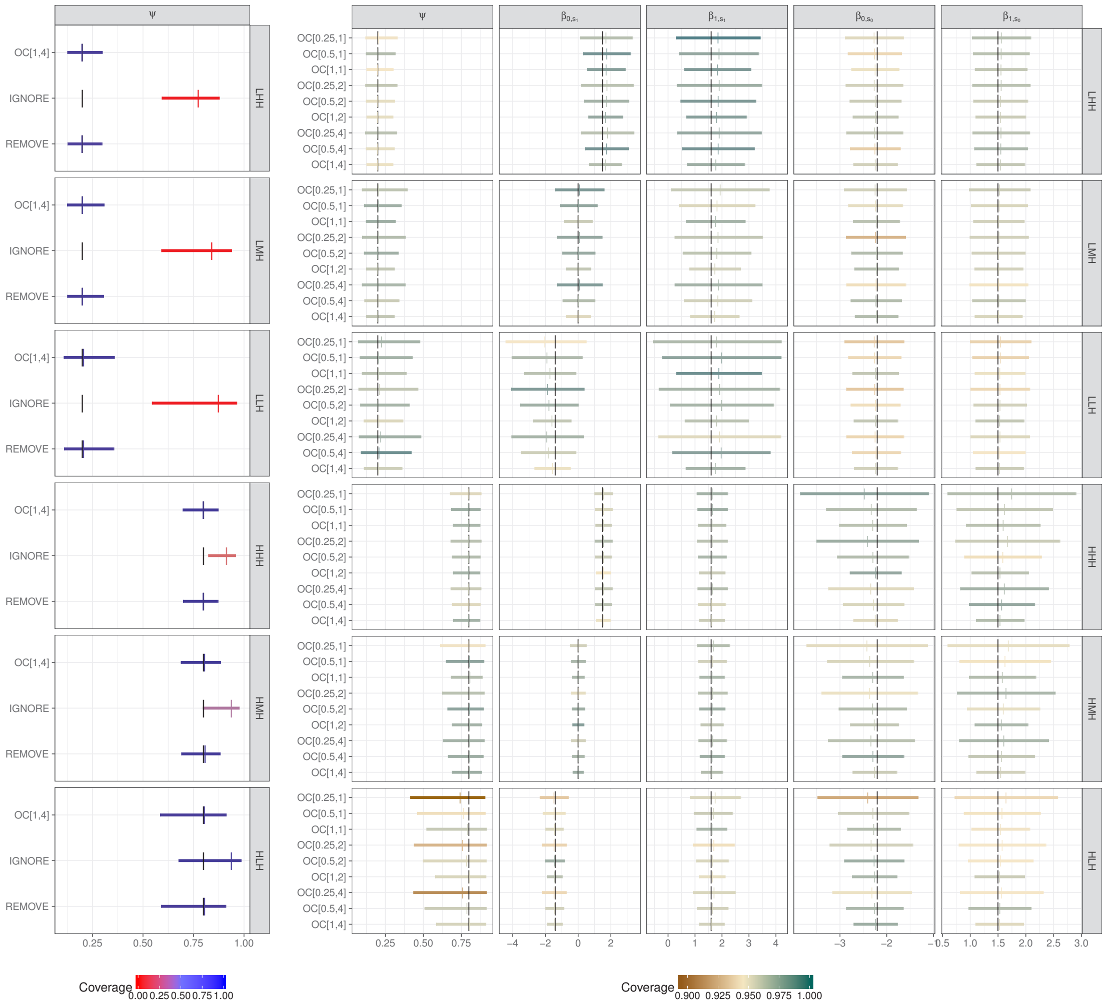

Figure S2.2: **Assumed data-generating parameter combinations for easier-to-misidentify species**: Left: Comparison of average approximate 95% CIs, bias (distance between assumed parameter combinations (shown as rows) used in data generation (black ticks) and average estimates (colored ticks), and coverage (0 = red, 1 = blue) for three approaches (*REMOVE*, *IGNORE*, and OC model fit to unambiguous data). Right: Comparison of the same results as those compared among the three models, but for the OC model applied to all confirmation designs. *Note that the coverage for the OC model confirmation design comparisons ranges from 0.9 (brown) to 1 (green).* Both: Three-letter row-labels indicate assumed occupancy (L = narrowly distributed, H = widely distributed), baseline detection (L = hard to detect, M = average, H = easy to detect), and baseline misidentification (L = hard to misidentify, H = easy to misidentify)

Convergence issues are shown in Figure **??** for all estimated parameters. There is an inverse relationship between the number of ML-estimation procedures that failed to converge and the amount of unambiguous data used in the approach for all parameters.

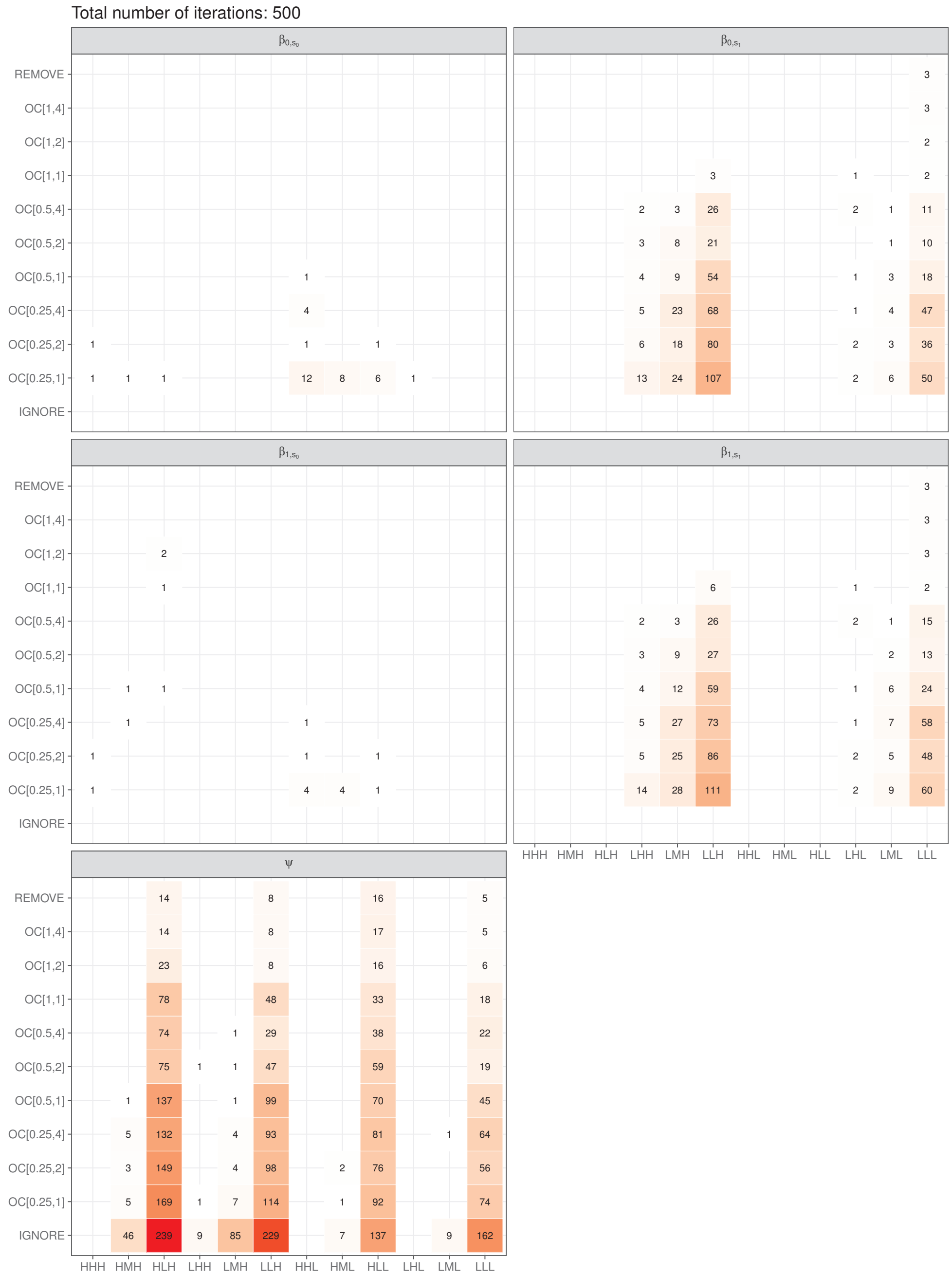

Figure S2.3: Heatmaps for all OC model parameters (panels) showing the number of iterations out of 500 that did not converge for the modeling approach taken (y-axis) and assumed parameter combination (x-axis). Three-letter row-labels indicate assumed occupancy (L = narrowly distributed, H = widely distributed), baseline detection (L = hard to detect, M = average, H = easy to detect), and baseline misidentification (L = hard to misidentify, H = easy to misidentify). The effort required from each confirmation design decreases from all visits to no confirmation down the y-axis (top to bottom). Color intensity increases with an increasing number of iterations that failed to converge.
