# Supplementary material for: Improving geographically extensive acoustic survey designs for modeling species occurrence with imperfect detection and misidentification
Source: Ecol Evol. 2018 May 20;8(12):6144–56. doi: 10.1002/ece3.4162 (PMC6024138; doi:10.1002/ece3.4162)
Supplement: Supplementary file 3 [file ECE3-8-6144-s003.pdf]

Supporting S3: `OCacoustic`

Designing and modeling geographically extensive bat acoustic surveys with imperfect detection and misidentification

Disclaimer: *This software has been approved for release by the U.S. Geological Survey (USGS). Although the software has been subjected to rigorous review, the USGS reserves the right to update the software as needed pursuant to further analysis and review. No warranty, expressed or implied, is made by the USGS or the U.S. Government as to the functionality of the software and related material nor shall the fact of release constitute any such warranty. Furthermore, the software is released on condition that neither the USGS nor the U.S. Government shall be held liable for any damages resulting from its authorized or unauthorized use.*

This document contains instructions for using `OCacoustic` to conduct custom simulation studies for investigating trade-offs in bias, precision, and coverage of OC model estimators among number of sites, number of visits, data-generating values, and modeling approaches: *IGNORE*, *REMOVE*, and the OC model applied to data from different confirmation designs. First, the package must be installed.

Installing `OCacoustic`

Download from BitBucket and install

To use `OCacoustic`, follow these two steps:

1. Download all files in the `OCacoustic` USGS BitBucket repository here (https://my.usgs.gov/bitbucket/users/kbanner_usgs.gov/repos/ip-092225/browse/DataS1).

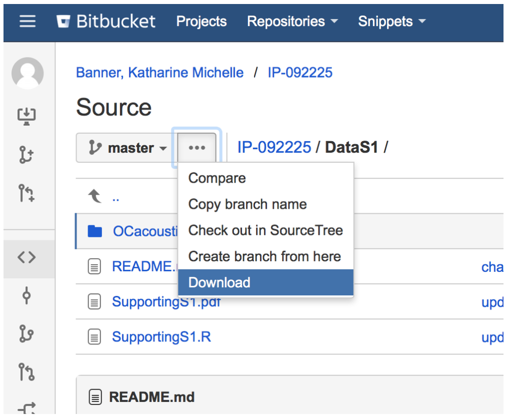

2. Set your working directory to the location of `OCacoustic` on your computer.
3. Run the following lines of code to load the package and its documentation.

```
# install the package and its dependent packages
devtools::install(".", dependencies = T)
```

4. To update `OCacoustic` unload the package with `unload(inst("OCacoustic"))` and repeat steps 1-3. Note that both functions, `inst` and `unload`, are in the `devtools` package.

`OCacoustic` uses functions from the following packages: `unmarked` (Fiske and Chandler 2011), `magrittr` (Bache and Wickham, n.d.), `dplyr` (Wickham and Francois 2016), `reshape2` (Wickham 2007), `roxygen2` (Wickham, Danenberg, and Eugster 2017), `latex2exp` (Meschiari 2015), `gridExtra` (Auguie 2016), and `ggplot2` (Wickham 2009). These packages will be loaded automatically by running `install_github`.

Designing and conducting simulation studies using `OCacoustic`

We encourage investigators to use pilot-data to obtain realistic data-generating values (parameter combinations) to use in their own custom simulation studies. Here, we provide step-by-step instructions for conducting such investigations.

Step 1: obtain a range of reasonable data-generating values

The function `oc_mle` fits the extended OC model specified by the function arguments. At a minimum, it requires a data frame in long format, `df_oc`, containing exactly four columns in

the following order:

- **Column 1:** `site` - a site ID for each observation
- **Column 2:** `visit` - a visit ID for each observation
- **Column 3:** `I_C` - The indicator variable specifying the confirmation design (e.g., if all visit-level detections are confirmed, `I_C` $= 1$ for all visits).
- **Column 4:** `obs_data` - the OC model response, which is a combination of ν and y_{ambig} values for the given confirmation design ($I(C)$).

An example of data in these format is provided in the package (`df_oc_epfu`). See the section titled *Formatting data for use in `oc_mle`* for instructions describing how to get from raw audio files to the response variable for the OC model.

```
# example data frame - bat species EPFU
head(df_oc_epfu)
```

```
##    site   revisit I_C obs_data
## 1 95426 revisit_1   1        0
## 2 96346 revisit_1   1        2
## 3 96350 revisit_1   1        0
## 4 96362 revisit_1   1        0
## 5 96805 revisit_1   1        0
## 6 96806 revisit_1   1        3
```

Running `oc_mle` with `df_oc` specified will fit the "null" model for occupancy (constant ψ for all sites, and constant s_0 and s_1 for all visits within sites).

```
# fit the extended oc model with constant
# occupancy and detection probabilties
fit_epfu <- oc_mle(df_oc = df_oc_epfu)

# view output - note: on the logit scale
fit_epfu
```

```
## $nlm_out
## $nlm_out$minimum
## [1] 210.637
##
## $nlm_out$estimate
## [1]  0.5941333  0.1159470 -2.1351564
```

```
##
## $nlm_out$gradient
## [1]  4.544631e-05 -2.188472e-05 -6.859313e-05
##
## $nlm_out$hessian
##           [,1]      [,2]     [,3]
## [1,] 12.707565  6.328725  0.00000
## [2,]  6.328725 26.461328  0.00000
## [3,]  0.000000  0.000000 21.46418
##
## $nlm_out$code
## [1] 1
##
## $nlm_out$iterations
## [1] 8
##
##
## $start_vals
## [1] "Tried 1 starting value(s) for parameter optimization"
##
## $summ
##               par        est        SE
## 1 (Intercept):psi  0.5941333 0.2988881
## 2 (Intercept):s_0 -2.1351564 0.2158454
## 3 (Intercept):s_1  0.1159470 0.2071257
##
## $iter
## [1] 1
##
## $par_names
## [1] "(Intercept):psi" "(Intercept):s_1" "(Intercept):s_0"
```

The function `ci_fun_oc` will compute approximate 95% (estimate ± 2 SE) CIs for each OC model parameter to provide ranges of plausible values for the species of interest. The argument `cov_idx` indicates which parameters to leave on the logit scale. If left unspecified, it will back-transform all CIs to the probability scale.

```r
# CIs on logit scale (lower, upper, est)
ci_fun_oc(fit_epfu, cov_idx = 1:3)
```

```
##                        lower      upper        est
## (Intercept):psi -0.003642955  1.1919096  0.5941333
## (Intercept):s_1 -0.298304484  0.5301984  0.1159470
## (Intercept):s_0 -2.566847291 -1.7034655 -2.1351564
```

Do not be alarmed by the warning message: `In nlm(p = theta, f = oc_lhood_covdf, df = dat_df, hessian = T) : NA/Inf replaced by maximum positive value`: This warning comes from the iterative process running under the hood of `nlm` which is used to find the minimum of the negative log likelihood (*nll*) for the OC model (ML-estimation). In the process of searching for the minimum, `nlm` encountered *nll* values that were too large to be handled by the computer. Since the values of the OC model parameters returning large *nll* values are not those that will return the minimum *nll* value (MLE), `nlm` assigns the largest *nll* value it has observed in the search to avoid an error. Because the OC model parameter values that produce the minimum value of the *nll* are the MLEs, this warning can be ignored. **Take heed to other warning messages and investigate them!**

To model heterogeneity in occupancy and detection probabilities, data for site- and visit-level covariates must be provided to the arguments `covs_site` and `covs_revisit` respectively. These data frames require *site* ID (for `covs_site`) and *site* and *visit* IDs (for `covs_revisit`) variables that match those in `df_oc`. A standard R formula specifying which covarites to use in each level of the model must also be specified (`site_form` and `rev_form`, respectively). A fake example data frame, `df_ex2` ($N = 100$, $J = 6$), and corresponding site- and visit-level covariates `cov_site2` and `cov_rev2` are built into the package to demonstrate fitting more complex covariate structures. `cov_site2` contains two site-level covariates, *clutter* (categorical with 4 categories) and *veg* (quantitative). `cov_rev2` contains two quantitative visit-level covariates *elev* and *calls*.

```r
# look at the data
head(df_ex2)
```

```
##   site revisit I_C obs_data
## 1    1       1   1        2
## 2    1       2   1        2
## 3    1       3   1        2
## 4    1       4   1        0
```

```
## 5     1       5   1        3
## 6     1       6   1        2
```

```r
head(cov_site2)
```

```
##   site clutter        veg
## 1    1       4  0.1690674
## 2    2       1 -0.4892017
## 3    3       2  1.3916152
## 4    4       3 -0.3028114
## 5    5       2 -1.0882969
## 6    6       1 -0.4509084
```

```r
head(cov_rev2)
```

```
##   site revisit       elev     calls
## 1    1       1  0.1343525 1.6785216
## 2    2       1 -0.4156935 1.0152141
## 3    3       1  1.2483523 1.5829940
## 4    4       1 -0.8942888 0.7297845
## 5    5       1 -0.1321642 1.1632461
## 6    6       1 -1.0710236 1.9342764
```

To model both site- and visit-level covariates with additive relationships between the covariates and the average *logit* of the detection probabilities, we specify `site_form = ~ clutter + veg` and `rev_form = ~ elev + calls` in `oc_mle`.

```r
# specify the mean structures and fit the models
oc_complex <- oc_mle(site_form = ~clutter + veg,
                     rev_form = ~ elev + calls,
                     df_oc = df_ex2,
                     covs_revisit = cov_rev2,
                     covs_site = cov_site2)

# look at confidence intervals - logit scale
n_pars <- length(oc_complex$nlm_out$estimate)
ci_fun_oc(oc_complex, cov_idx = c(1:n_pars))
```

```
##                        lower      upper       est
## (Intercept):psi  0.510912818  2.11817587  1.3145443
```

```
## clutter2:psi    -0.645780869  2.97936495  1.1667920
## clutter3:psi    -1.394358529  1.05921335 -0.1675726
## clutter4:psi     0.345974048  4.10071374  2.2233439
## veg:psi         -2.353496722 -0.66059253 -1.5070446
## (Intercept):s_1  1.246736077  1.75722233  1.5019792
## elev:s_1        -0.437425001  0.04358574 -0.1969196
## calls:s_1        0.154895089  0.66472111  0.4098081
## (Intercept):s_0 -0.045429972  0.29952160  0.1270458
## elev:s_0        -0.001087653  0.33411941  0.1665159
## calls:s_0        0.464271800  0.82502538  0.6446486
```

Any standard formula can be used to specify the mean structure (e.g., `-1` for no intercept, `a*b` for additive terms and an interaction between the covariates `a` and `b`). For more details, refer to the help file by running `? oc_mle` in the console.

Step 2: compare modeling approaches and confirmation designs

Simulating data under the OC model

The function `oc_sim_gen` is a simulation wrapper that first calls `oc_datasim` to generate data under the OC model for a specified sample size (`N` = N, `J` = J), set of data-generating values (`site_psi` = parameters related to ψ, `rev_s0` = parameters related to false-detection s_0, `rev_s1` = parameters related to true-detection s_1), model form (`site_form` = R formula specifying the mean structure for site-level covariates [`covs_site`], `rev_form` = R formula specifying the mean structure for visit-level covariates [`covs_revisit`]), set of confirmation designs (`prop_sites` = a vector of p values, `num_revisit` = a vector of d values), and two standard occupancy models - REMOVE and IGNORE (data generated as described in Section 2.3). Covariate data used in the data simulation are re-sampled (by row) from `covs_revisit` and `covs_site` until the desired number of rows is achieved for each set of covariates (N for `covs_site`, $N \times J$ for `covs_revisit`). Then, `oc_sim_gen` fits datasets from each confirmation design to the OC model using `oc_mle`, and it fits datasets for the REMOVE and IGNORE approaches to a standard occupancy model using `occu` (Fiske and Chandler 2011). For each approach (e.g., IGNORE, $OC_{1,2}$, etc.) the data, estimates, confidence intervals (CIs), error codes, and capture statuses (data generating value in CI or not) are saved for each parameter. This process constitutes one realization, it is repeated a user-specified number of times (`n_sims`).

Here, we demonstrate the use of `oc_sim_gen` using small (`n_sims = 10`) simulations for two sets of data-generating values with different occupancy probabilities (`site_psi = 1.4` or `site_psi = -1.35` and `rev_s1 = c(0, 1.6)`, `rev_s0 = c(-2.2, 1.5)`). *Note that the data-generating values must be provided on the logit scale.* We assume a sampling design with `N = 50` sites and `J = 6` visits. We consider nine confirmation designs (all combinations of `prop_sites = c(1,0.5,0.25)` and `num_revisit = c(1,3,6)`). The dataset, `covs_sim` contains the $log(K+1)$ values observed in the Oregon data (built into `OCacoustic`). The argument `std` takes on a logical value `TRUE` if continuous covariates are to be re-scaled to have mean $= 0$ and sd $= 1$ prior to model fitting. The arguments `psi_prob` and `det_prob` are by default `FALSE`, but when set to `TRUE` will track results on the probabiltiy scale for the occupancy parameters and detection parameters, respectively.

```
# covs_sim is a data.frame with the log(K+1) values
head(covs_sim)

# specify inputs and conduct the simulation; this may
# take a little over a minute. Progress bars indicate
# progress of each iteration
HMH_50 <- oc_sim_gen(n_sims = 10,
                     N = 50,
                     J = 6,
                     site_form = ~ 1,
                     rev_form = ~ K,
                     occu_form = ~ K ~ 1,
                     site_psi = 1.4,
                     rev_s0 = c(-2.2, 1.5),
                     rev_s1 = c(0, 1.6),
                     prop_sites = c(1,0.5,0.25),
                     num_revisit = c(1,3,6),
                     covs_revisit = covs_sim,
                     std = TRUE,
                     psi_prob = TRUE)

# low occupancy (~ 0.2 on prob scale)
LMH_50 <- oc_sim_gen(n_sims = 10,
                     N = 50,
                     J = 6,
```

```
                        site_form = ~ 1,
                        rev_form = ~ K,
                        occu_form = ~ K ~ 1,
                        site_psi = -1.35,
                        rev_s0 = c(-2.2, 1.5),
                        rev_s1 = c(0, 1.6),
                        prop_sites = c(1,0.5,0.25),
                        num_revisit = c(1,3,6),
                        covs_revisit = covs_sim,
                        std = TRUE,
                        psi_prob = TRUE)
```

Again, do not be alarmed by the warning message: `In nlm(p = theta, f = oc_lhood_covdf, df = dat_df, hessian = T) : NA/Inf replaced by maximum positive value` (see *Step1: obtain a range of reasonable data-generating values*).

`HMH_50` includes information for each iteration of the simulation:

- `$ci_list` a list (one element for each modeling approach) of lists (length = `n_sims`) containing CIs for each OC model parameter for each iteration.

```
# See which approach corresponds to
# each element of $ci_list
names(HMH_50$ci_list)
```

```
##  [1] "OC[1,6]"    "OC[0.5,6]"  "OC[0.25,6]" "OC[1,3]"    "OC[0.5,3]"
##  [6] "OC[0.25,3]" "OC[1,1]"    "OC[0.5,1]"  "OC[0.25,1]" "REMOVE"
## [11] "IGNORE"
```

```
# Results from the OC model applied to unambiguous
# data only (OC[1,6]) are stored in the first element
# of $ci_list ($ci_list[[1]]).
HMH_50$ci_list[[1]][[1]]
```

```
##                  minus_2SE   plus_2SE         est
## (Intercept):psi  0.7226329  0.9370695  0.86165962
## (Intercept):s_1 -0.3125312  0.3405324  0.01400063
## K:s_1            1.2756725  2.1067850  1.69122873
## (Intercept):s_0 -2.4910738 -1.5700444 -2.03055910
```

```
## K:s_0             1.2038476  2.0959354  1.64989152
```

- `$capture_list` a list (one element for each vetting scenario) containing a matrix indicating whether the data-generating values for the OC model parameters were captured by each iterations' estimated CI (iterations indexed by rows, parameters by columns)

```r
# See if data-generating values were captured
HMH_50$capture_list[[1]][1, ]
```

```
## (Intercept):psi (Intercept):s_1           K:s_1 (Intercept):s_0
##            TRUE            TRUE            TRUE            TRUE
##           K:s_0
##            TRUE
```

- `$est_list` a list (one element for each modeling scenario) containing matrices storing the estimated parameter values (cols) for each iteration (row)

```r
# Look at just the estimates
HMH_50$est_list[[1]][1, ]
```

```
## (Intercept):psi (Intercept):s_1           K:s_1 (Intercept):s_0
##      0.86165962      0.01400063      1.69122873     -2.03055910
##           K:s_0
##      1.64989152
```

- `$error` a list (one element for each approach) of vectors storing a 0, 1, or 2, indicating whether or not the likelihood could be maximized at the randomly generating starting values (0 = yes, 1 = yes, but Hessian was singular, 2 = no, randomly-generated starting values) for each iteration.
- `$error_se` a list (one element for each approach) of vectors storing a 0 or 1, indicating whether or not the Hessian could be inverted to obtain SEs (0 = yes, 1 = no).

```r
# did the starting values result in convergence? (first iteration)
HMH_50$error[[1]][1]
```

```
## [1] 0
```

```r
# was the hessian invertible (first iteration)
HMH_50$error_se[[1]][1]
```

```
## [1] 0
```

- `$data` the data used in the data-generation and model fitting for each iteration

```r
# what is stored for each iteration?
names(HMH_50$data[[1]])
```

```
## [1] "all_dat"      "occu_dat"     "obs_data"     "D_A"
## [5] "D_C"          "covs_revisit" "covs_site"    "occu_revisit"
## [9] "occu_site"
```

```r
# the data frame used to create all OC datasets (occu_dat)
head(HMH_50$data[[1]]$occu_dat)
```

```
##   site revisit nu y_ambig y_conf           K
## 1    1       1  2       1      1 -0.12059174
## 2    1       2  0       0      0 -1.33181035
## 3    1       3  0       0      0 -1.33181035
## 4    1       4  0       0      0 -0.38929907
## 5    1       5  2       1      1  0.05640126
## 6    1       6  0       0      0 -1.33181035
```

```r
# a list of all data frames used in the OC model fits (obs_data)
# datasets for each of the confirmation designs
# are stored in HMH_50$data[[1]]$obs_data
# for the unambiguous only data
head(HMH_50$data[[1]]$obs_data[[1]])
```

```
##   site revisit I_C obs_data
## 1    1       1   1        2
## 2    1       2   1        0
## 3    1       3   1        0
## 4    1       4   1        0
## 5    1       5   1        2
## 6    1       6   1        0
```

```r
# Deteciton history used in IGNORE (D_A)
head(HMH_50$data[[1]]$D_A)
```

```
##      [,1] [,2] [,3] [,4] [,5] [,6]
## [1,]    1    0    0    0    1    0
## [2,]    1    0    0    1    0    1
```

```
## [3,]    1    0    0    1    1    0
## [4,]    0    0    0    0    0    0
## [5,]    1    1    0    1    0    0
## [6,]    1    0    1    1    1    0
```

```
# Deteciton history used in REMOVE (D_C)
head(HMH_50$data[[1]]$D_C)
```

```
##      [,1] [,2] [,3] [,4] [,5] [,6]
## [1,]    1    0    0    0    1    0
## [2,]    1    0    0    1    0    1
## [3,]    1    0    0    1    1    0
## [4,]    0    0    0    0    0    0
## [5,]    1    1    0    0    0    0
## [6,]    1    0    1    1    1    0
```

```
# visit-level covariates for OC model (covs_revisit)
head(HMH_50$data[[1]]$covs_revisit)
```

```
##     site revisit           K
## 1      1       1 -0.12059174
## 51     1       2 -1.33181035
## 101    1       3 -1.33181035
## 151    1       4 -0.38929907
## 201    1       5  0.05640126
## 251    1       6 -1.33181035
```

```
# site-level covariates for IGNORE AND REMOVE (occu_revisit)
head(HMH_50$data[[1]]$occu_revisit)
```

```
##             K
## 1 -0.12059174
## 2 -1.33181035
## 3 -1.33181035
## 4 -0.38929907
## 5  0.05640126
## 6 -1.33181035
```

- `$truth` the data-generating values used for the OC model parameters in a vector

```r
# HMH data-generating values
round(HMH_50$truth, digits = 2)
```

```
## [1]  0.8  0.0  1.6 -2.2  1.5
```

Visualize results for each individual case considered using `sim_process`:

```
# Summarize results
hmh50_out <- sim_process_gen(HMH_50)
```

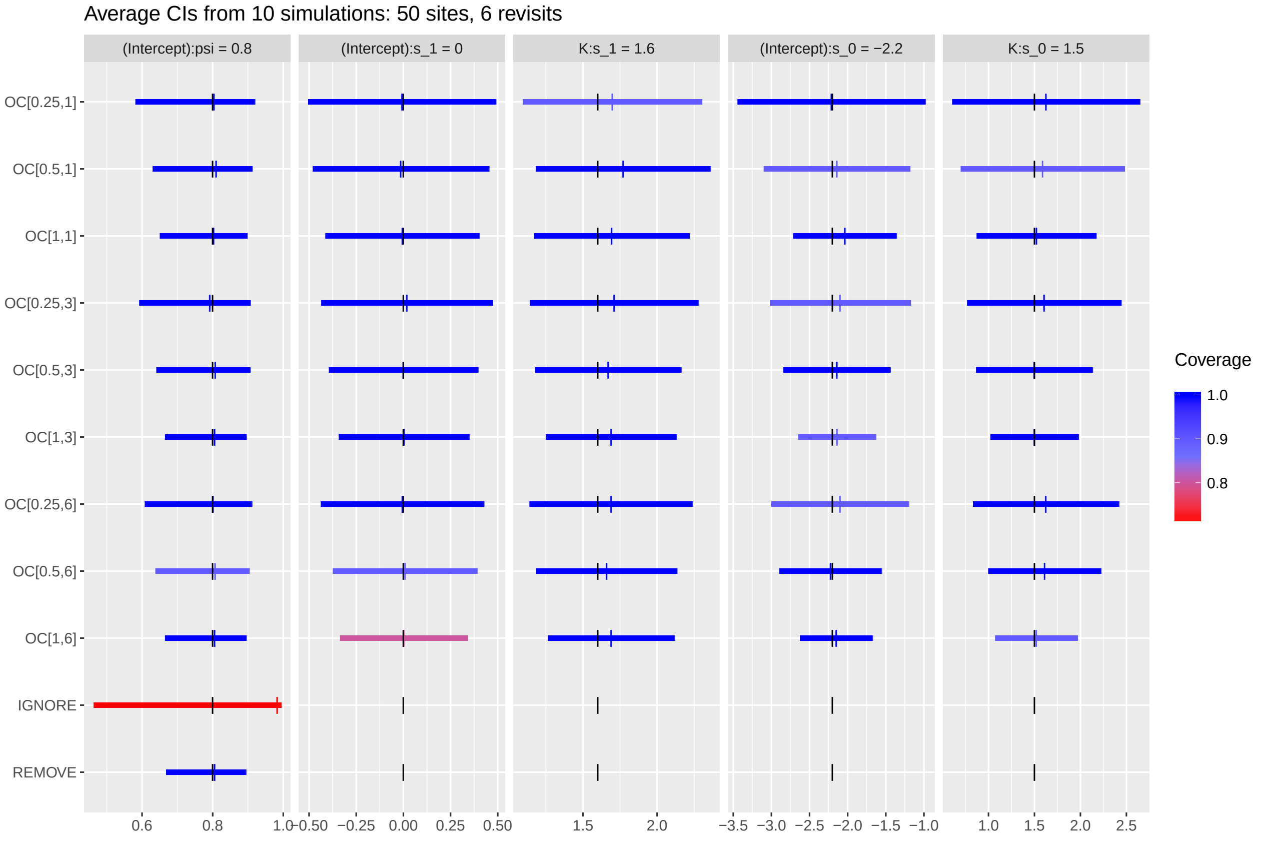


```
# Summarize results
lmh50_out <- sim_process_gen(LMH_50)
```

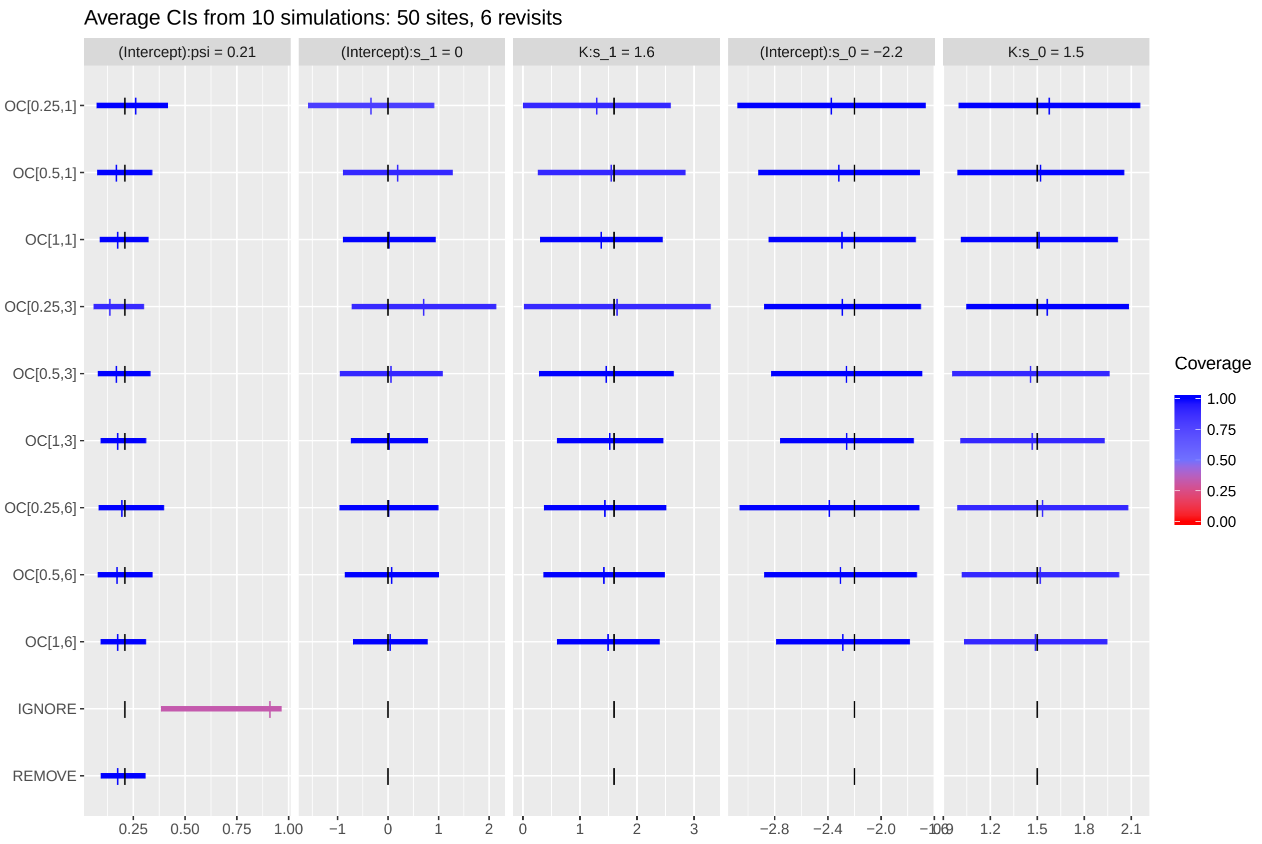
Average CIs from 10 simulations: 50 sites, 6 revisits
(Intercept):psi = 0.21
(Intercept):s_1 = 0
K:s_1 = 1.6
(Intercept):s_0 = −2.2
K:s_0 = 1.5
OC[0.25,1]
OC[0.5,1]
OC[1,1]
OC[0.25,3]
OC[0.5,3]
OC[1,3]
OC[0.25,6]
OC[0.5,6]
OC[1,6]
IGNORE
REMOVE
0.25 0.50 0.75 1.00
−1 0 1 2
0 1 2 3
−2.8 −2.4 −2.0
1.2 1.5 1.8 2.1
Coverage
1.00
0.75
0.50
0.25
0.00

Compare results among the two standard occupancy models and the OC model fit to unambiguous data only using `FNOC_psi_compare`. The `oc_ref` argument specifies which OC modeling scenario corresponds to the confirmation design requiring the most effort (in this case `"OC[1,6]"`).

```
# Create a list of sim results, elements MUST
# be named
sim50_list <- list("HMH_50" = hmh50_out$ci_summ,
                   "LMH_50" = lmh50_out$ci_summ)

# Compare 3 common approaches FN- to OC
psi_compare <- FNOC_psi_compare(sim50_list, fontsize = 16,
                                oc_ref = "OC[1,6]")
```

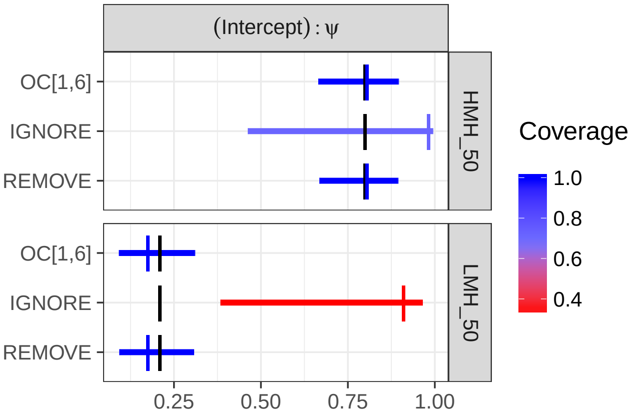

Compare vetting scenarios using `oconly_compare_gen`:

```
# Compare confirmation designs
oc_compare <- oconly_compare_gen(sim50_list, fontsize = 16)
```

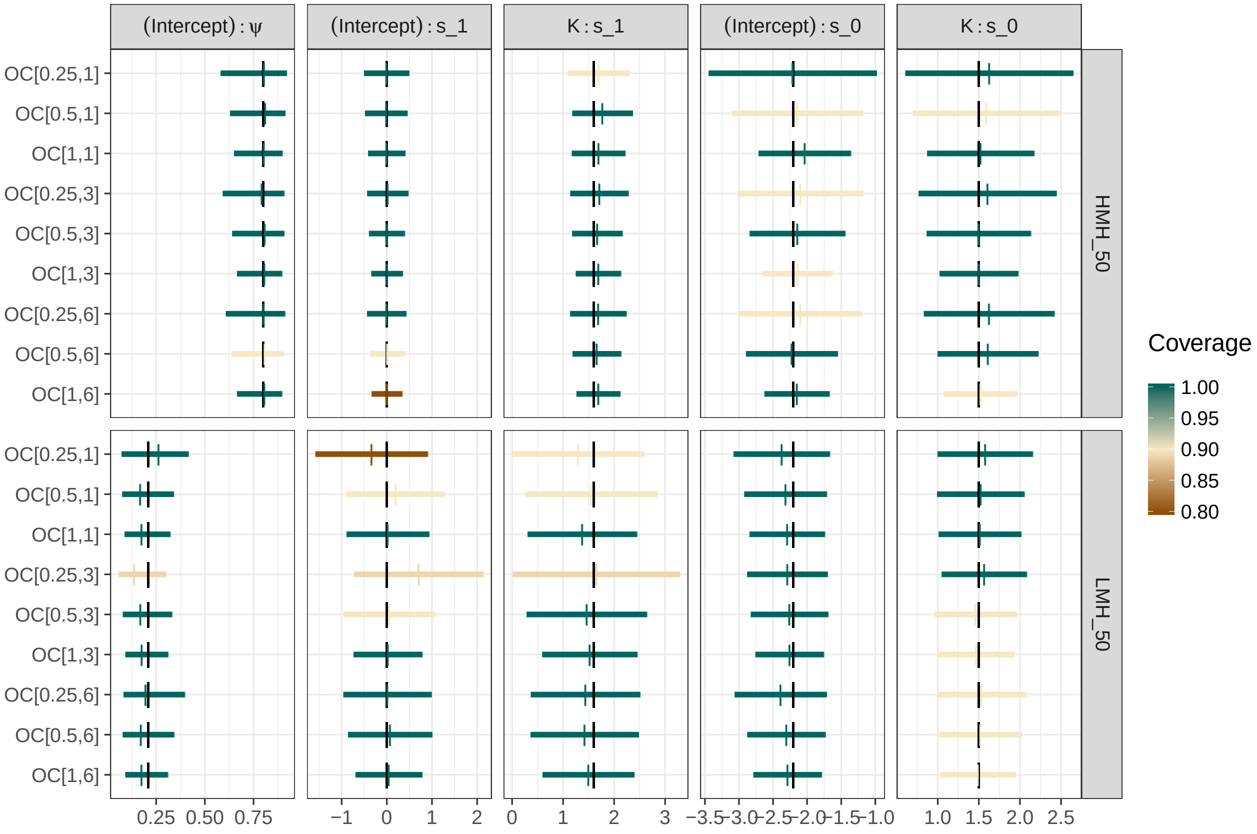

Check convergence issues and create a plot like Figure 5 and Figure B.3 using `converge_plot`.

```
# create a list of iterations that were excluded from
# summaries
iter_exclude <- list("HMH" = hmh50_out$dat_huge,
                     "LMH" = lmh50_out$dat_huge)
# grab par levels
par_levels <- dimnames(HMH_50$ci_list[[1]][[1]])[[1]]

# grab method approaches
methods <- names(HMH_50$ci_list)

# make the diagnositc plot if any rows were removed
if(sum(sapply(iter_exclude,nrow)) > 0) {
  rm_plot50 <- converge_plot(iter_exclude, num_sims = 10,
```

```
                                              methods =  methods,
                                              par_levels = par_levels,
                                              fontsize = 16)
}
```

All functions are fully documented. For more details about these functions use the documentation (e.g., `? oconly_compare_gen`).

oconly_compare_gen {OCacoustic} R Documentation

Compare all OC model confirmation designs

Description

Compare all OC model confirmation designs

Usage

```
oconly_compare_gen(sim_runs, fontsize = 14)
```

Arguments

`sim_runs`	a list of simulation results, each element being the CI summary (first element) of `sim_process`. Note that each element of the list must be named.
`fontsize`	a number specifying the fontsize for the plot

Value

A plot and `data.frame` displaying/providing the values for the endpoints of the average 95% confidence intervals, average estimates, data generating values, and coverage rates for each OC model parameter under each confirmation design investigated in `sim_runs`.

In the manuscript, we accounted for heterogeneity in the detection probabilities (s_0/s_1) using a transformation of the number of bat calls (K) at visit locations ($log(K+1)$) as a linear predictor for the *logit* of the detection probabilities, after which, we assumed constant ψ. Conducting a simulation with 500 iterations for a survey with $N = 84$ sites, $J = 4$ visits and considering modeling approaches ($IGNORE$, $REMOVE$, $OC_{1,4} \ldots, OC_{0.25,1}$), took up to an hour-and-a-half on a MacBook Pro with 2.6 GHz Intel Core i7 processor and 16 GB of memory. The exact time for custom simulations will depend on the complexity of the mean structure specified, the data-generating values, and the confirmation designs explored.

Generating confirmation designs

Suppose we conducted a full simulation study for this study design and chose a confirmation design based on our results. Now suppose we conducted a survey and retrieved ambiguous detection/non-detection data from visiting 50 sites in 6 locations in space/time. Now, we can use `conf_design_fun` to randomly select visits to confirm given our chosen confirmation design. This function takes on the ambiguous detection data as an input `y_auto` to ensure visits where the focal species was detected are selected for verification before visits resulting in non-detections.

```
# we'll use auto detection data from one of the
# iterations of the simulation, but these data would
# be observed in the field during a real study
y_auto <- HMH_50$data[[1]]$D_A
head(y_auto)
```

```
##      [,1] [,2] [,3] [,4] [,5] [,6]
## [1,]    1    0    0    0    1    0
## [2,]    1    0    0    1    0    1
## [3,]    1    0    0    1    1    0
## [4,]    0    0    0    0    0    0
## [5,]    1    1    0    1    0    0
## [6,]    1    0    1    1    1    0
```

```
vm <- conf_design_fun(y_auto = y_auto, N = 50, J = 6,
                      prop_sites = 1,
                      num_revisit = 3)
head(vm)
```

```
##      [,1] [,2] [,3] [,4] [,5] [,6]
## [1,]    1    0    0    0    1    1
## [2,]    1    0    0    1    0    1
## [3,]    1    0    0    1    1    0
## [4,]    1    0    1    0    0    1
## [5,]    1    1    0    1    0    0
## [6,]    1    0    1    1    0    0
```

Suppose, in collecting the data, there were equipment malfunctions or logistical constraints and we are missing data from some of the visit locations. The function `conf_design_applied`

can be used to accommodate this situation. However, it should be noted that the simulation was done assuming there would be complete sampling and missing data could affect the choice of confirmation design. Run through the following code to investigate this function.

```
idx_na <- sample(x = 1:300, size = 50)
y_auto[idx_na] <- NA
head(y_auto)
```

```
##      [,1] [,2] [,3] [,4] [,5] [,6]
## [1,]    1    0   NA    0    1    0
## [2,]    1    0   NA    1    0   NA
## [3,]    1    0    0    1   NA   NA
## [4,]    0    0    0    0    0    0
## [5,]    1    1    0    1    0    0
## [6,]    1   NA    1    1    1    0
```

```
vmat <- y_auto
vmat[!is.na(vmat)] <- 1
head(vmat)
```

```
##      [,1] [,2] [,3] [,4] [,5] [,6]
## [1,]    1    1   NA    1    1    1
## [2,]    1    1   NA    1    1   NA
## [3,]    1    1    1    1   NA   NA
## [4,]    1    1    1    1    1    1
## [5,]    1    1    1    1    1    1
## [6,]    1   NA    1    1    1    1
```

```
vm_na <- conf_design_applied(y_ambig = y_auto, nu = vmat, N = 50, J = 6,
                             prop_sites = 1, num_revisit = 3)
head(vm_na)
```

```
##      [,1] [,2] [,3] [,4] [,5] [,6]
## [1,]    1    1   NA    0    1    0
## [2,]    1    0   NA    1    1   NA
## [3,]    1    1    0    1   NA   NA
## [4,]    1    1    0    0    1    0
## [5,]    1    1    0    1    0    0
## [6,]    1   NA    0    1    1    0
```

Formatting data for use in `oc_mle`

Step 1: clean and process

Raw audio files can be stored in a database, where deployments can be queried. The data we used in the manuscript were collected in Oregon, USA. These data are stored in an Access database maintained by the National Park Service (NPS). A specific query to this database will result in a .csv `allOregonData`, which are included in `OCacoustic` as a `data.frame`. Use `head`, `tail`, and `summary` to explore the format of these data.

Output from a database query will often require some cleaning. We wrote a custom function for the *Manul IDs* observed in our data and the Pacific North West classifier from Sonobat, `PNW_process`, that ensures *Auto ID* labels are consistent with *Manual ID* labels. A function like this will be project specific, so we provide ours as an example for practitioners to use when writing their own. Run `PNW_process` in the R console to view the function.

```
dat <- PNW_process(data = allOregonData)
```

The data frame returned will have 28 columns for the following variables (in the following order):

```
pander::pander(names(dat))
```

owner, *hab_broad*, *hab_local*, *water_type*, *year*, *deploy_date*, *night*, *park_code*, *park_name*, *grid_cell*, *detector*, *classifier*, *filename*, *spp_acc*, *man_ID1*, *man_ID2*, *notes*, *clutter_pct*, *clutter_type*, *clutter_dist*, *mic_height*, *datum*, *lat*, *long*, *elev*, *quad*, *detect_agg* and *man_IDadj*

Site and visit information in columns 1-11

- owner: Land ownership
- hab_broad: Category for broad habitat
- hab_local: Category for local habitat
- water_type: Category for water type, if any
- year: Year
- deploy_date: Deployment date
- night: Date of survey night
- park_code: Park code
- park_name: Park name
- grid_cell: Unique identifier for site
- detector: Unique identifier for visit location prior to data cleaning

Classification information in colums 12-16

- classifier: Classification software
- filename: File-name from classification software (unique identifier for call file)
- spp_acc: Primary classification from *Auto ID*
- man_ID1: Primary classification from Manual verification prior to cleaning
- man_ID2: Secondary classification from Manual verification (not used in analysis)

Covariate information in columns 17-25

- notes: Field notes - observation-level
- clutter_pct: Category for percent clutter (visit-level)
- clutter_type: Category for clutter type (site-level)
- clutter_dist: Distance to clutter (visit-level)
- mic_height: Microphone height (visit-level)
- datum: Datum
- lat: Latitude (visit-level)
- long: Longitude (visit-level)
- elev: Elevation (visit-level)

Cleaned variables in colums 26-28

- quad: Quadrant identifier for detector deployment (i.e., if 2 were placed in NW: NW1, NW2, they both receive NW. See Appendix A).
- detect_agg: Cleaned unique identifier for visits
- man_IDadj: Cleaned *Manual ID*

Variables without information should be populated by `NA`s. Data frames with the variables described above can be further summarized for a single focal species using `format_query`. An error message will be returned if the data are not in the correct format and a warning message will be returned if no Auto IDs detect the focal species.

```
# try with allOregonData - wrong format
format_query(allOregonData)
```

```
## Error in format_query(allOregonData): Data must be formatted with columns for the 28
##          in the help file - ORDER MATTERS! use ? format_query
##          for more information
```

```
# try for Coto, an unestablished species with no Auto IDs
coto_data <- format_query(dat, spp = "Coto")
```

```
## Warning in format_query(dat, spp = "Coto"): species not detected, make sure argument
##                 is correct. If correct, this species was not
##                 detected
```

```
# try for Epfu, a widely-distributed spp
epfu_data <- format_query(dat, spp = "Epfu")

# det hist matrix for Auto ID $D_ambig
epfu_autoD <- epfu_data$D_ambig
head(epfu_autoD)
```

```
##       revisit_1 revisit_2 revisit_3 revisit_4
## 95426         0         0         0         0
## 96346         4        NA        NA        NA
## 96350         0         0         0        NA
## 96362         0         0         0        NA
## 96805         0        NA        NA        NA
## 96806        39        66        NA        NA
```

```
# y_ambig from auto IDs for IGNORE
epfu_y_ambig <- epfu_autoD
epfu_y_ambig[epfu_y_ambig > 0] <- 1

# det hist matrix for Manual ID $D_conf
epfu_manD <- epfu_data$D_conf
head(epfu_manD)
```

```
##       revisit_1 revisit_2 revisit_3 revisit_4
## 95426         0         0         0         0
## 96346         2        NA        NA        NA
## 96350         0         0         0        NA
## 96362         0         0         0        NA
## 96805         0        NA        NA        NA
## 96806         3         1        NA        NA
```

```
# y_confirmed from auto IDs for IGNORE
epfu_y_conf <- epfu_manD
epfu_y_conf[epfu_y_conf > 0] <- 1
```

```r
# proxy for quality in $K
epfu_autoK <- epfu_data$K
head(epfu_autoK)
```

```
##       revisit_1 revisit_2 revisit_3 revisit_4
## 95426        93         8         4         0
## 96346        13        NA        NA        NA
## 96350         5         0        19        NA
## 96362         0         0        37        NA
## 96805        42        NA        NA        NA
## 96806       337       787        NA        NA
```

```r
# summary of all observations in a data frame
epfu_det_df <- epfu_data$call_sum
head(epfu_det_df)
```

```
## # A tibble: 6 x 7
## # Groups:   grid_cell [3]
##   grid_cell location raw_calls n_spp_auto n_spp_conf n_confirmed bat_calls
##       <int> <chr>        <int>      <dbl>      <dbl>       <int>     <dbl>
## 1     95426 95426_NE       352       0          0             38     93.0
## 2     95426 95426_NW        14       0          0              8      8.00
## 3     95426 95426_SE         6       0          0              5      4.00
## 4     95426 95426_SW         5       0          0              1      0
## 5     96346 96346_NW        59       4.00       2.00          16     13.0
## 6     96350 96350_NE         6       0          0              4      5.00
```

The final step in the data formatting process is to obtain the ν values for the OC model. The function `obs_conf_data` does this. It requires the cleaned data, the focal species, and the detection history matrix for the *Auto IDs* (one of the objects returned by `format_query`).

```r
# format data for OC model
oc_epfu <- obs_conf_data(dat, spp = "Epfu",
                         D_ambig = epfu_autoD)
```

```
## Auto ID labels must match Manual ID labels! It is okay if non-species IDs do not matc
##  Auto Ids printed above Manual IDs below:
##  A_IDs: Anpa Epfu Euma Laci Lano Myca Myci Myev Mylu Myth Myvo Myyu Pahe Tabr
##  M_IDs: Anpa Epfu Euma Laci Lano Myca Myci Myev Mylu Myth Myvo Myyu other Pahe Tabr
```

```
##
```

```r
# detection history matrix of nu-values
epfu_unambig <- oc_epfu$nu
head(epfu_unambig)
```

```
##       revisit_1 revisit_2 revisit_3 revisit_4
## 95426         0         0         0         0
## 96346         2        NA        NA        NA
## 96350         0         0         0        NA
## 96362         0         0         0        NA
## 96805         0        NA        NA        NA
## 96806         3         2        NA        NA
```

```r
# Check the 2 from site 96346 look at individual call files
subset(oc_epfu$check,
       detect_agg == "96346_NW")
```

```
## # A tibble: 16 x 7
## # Groups:   grid_cell [1]
##    grid_cell detect_agg spp_acc man_IDadj man_ID1   auto vetted
##        <int> <chr>      <fct>   <fct>     <fct>    <dbl>  <dbl>
##  1     96346 96346_NW   <NA>    other     LoF       0      0
##  2     96346 96346_NW   Epfu    Epfu      EPFU      1.00   1.00
##  3     96346 96346_NW   Lano    Lano      Lano      0      0
##  4     96346 96346_NW   <NA>    other     Myotis    0      0
##  5     96346 96346_NW   <NA>    other     Myotis40  0      0
##  6     96346 96346_NW   <NA>    Tabr      TABR      0      0
##  7     96346 96346_NW   Epfu    Epfu      EPFU      1.00   1.00
##  8     96346 96346_NW   Mylu    Mylu      Mylu      0      0
##  9     96346 96346_NW   Mylu    Mylu      Mylu      0      0
## 10     96346 96346_NW   Mylu    Mylu      Mylu      0      0
## 11     96346 96346_NW   Mylu    Mylu      Mylu      0      0
## 12     96346 96346_NW   <NA>    other     Myotis50  0      0
## 13     96346 96346_NW   <NA>    Mylu      MYLU      0      0
## 14     96346 96346_NW   <NA>    other     LoF       0      0
## 15     96346 96346_NW   <NA>    other     LoF       0      0
## 16     96346 96346_NW   Mylu    Mylu      Mylu      0      0
```

```r
# this visit gets assigned a 2 ("all true")
subset(oc_epfu$summary,
       detect_agg == "96346_NW")
```

```
## # A tibble: 1 x 10
##   detect_agg grid_cell auto_sum man_sum num_vetted non_detect_agree
##   <chr>          <int>    <dbl>   <dbl>      <int>            <int>
## 1 96346_NW       96346     2.00    2.00         16               14
## # ... with 4 more variables: false_neg <int>, false_pos <int>,
## #   true_pos <int>, v <dbl>
```

Step 2: create data frames for `oc_mle`

To create `df_oc` for the Oregon data, we can use the output from `obs_conf_data` and `format_query`.

```r
# If available, row and column names of the detection
# history will populate the names of the site and visit
# fields. The values will be given the name given
# to "var_name"
df_epfu <- matrix_to_df(epfu_unambig,
                        var_name = "obs_data")
head(df_epfu)
```

```
##    site   revisit obs_data
## 1 95426 revisit_1        0
## 2 96346 revisit_1        2
## 3 96350 revisit_1        0
## 4 96362 revisit_1        0
## 5 96805 revisit_1        0
## 6 96806 revisit_1        3
```

```r
# Create the I(C_{p,d}) variable for the confirmation matrix,
# in this example each visit-level detection was confirmed,
# so prop_sites = 1 and num_revisit = 4 (all)
I_C <- conf_design_applied(epfu_y_ambig, nu = epfu_unambig,
                           N = 84, J = 4, prop_sites = 1,
```

```
                                              num_revisit = 4)
# make it a data frame
I_Cdf <- matrix_to_df(I_C, var_name = "I_C")
head(I_Cdf)
```

```
##    site   revisit I_C
## 1 95426 revisit_1   1
## 2 96346 revisit_1   1
## 3 96350 revisit_1   1
## 4 96362 revisit_1   1
## 5 96805 revisit_1   1
## 6 96806 revisit_1   1
```

```
# make the covariates a data frame
covs <- matrix_to_df(epfu_autoK, var_name = "K")
covs$K <- log(covs$K + 1)
covs$K <- (covs$K - mean(covs$K, na.rm = T))/sd(covs$K, na.rm = T)
covs <- covs[complete.cases(covs), ]

# create the data frame: first four columns must be site, visit,
# I_C, and then covs
df_oc <- dplyr::full_join(I_Cdf, df_epfu,
                              by = c("site", "revisit"))

# get rid of NA values - don't contribute to l-hood
df_oc <- df_oc[-which(is.na(df_oc$obs_data)),]
head(df_oc)
```

```
##    site   revisit I_C obs_data
## 1 95426 revisit_1   1        0
## 2 96346 revisit_1   1        2
## 3 96350 revisit_1   1        0
## 4 96362 revisit_1   1        0
## 5 96805 revisit_1   1        0
## 6 96806 revisit_1   1        3
```

```r
# Get the K-values from the observed data
covs <- matrix_to_df(epfu_autoK, var_name = "K")
covs$K <- log(covs$K + 1)

# center and scale
covs$K <- (covs$K - mean(covs$K, na.rm = T))/sd(covs$K, na.rm = T)

# get rid of NAs because they are missing
covs <- covs[complete.cases(covs), ]
```

Step 3: fit and compare approaches for many species

We ignored five of the sites that were sampled for a different purpose than the other seventy-nine sites, these five sites are listed in the vector `gc_drop`.

```r
# disregard 5 sites (grid cells)
gc_drop <- c(99569, 115792, 108830, 100037,  100038)

# drop those sites
idx_drop <- which(df_oc$site %in% gc_drop)
df_oc <- df_oc[-idx_drop, ]

# drop those sites
idx_covs <- which(covs$site %in% gc_drop)
covs <- covs[-idx_covs, ]
```

Fit the model

```r
# use oc_mle
fit_oc_epfu <- oc_mle(rev_form = ~ K,
                      covs_revisit = covs,
                      df_oc = df_oc)

# CI with psi on prob scale
ci_fun_lhood_nlm(fit_oc_epfu$nlm_out, cov_idx = c(2:5))
```

```
##                                      est
```

```
## [1,]  0.6284480  0.9223245  0.8175686
## [2,] -1.1655408 -0.1580256 -0.6617832
## [3,]  1.5443009  2.8443625  2.1943317
## [4,] -3.5157106 -2.1145179 -2.8151143
## [5,]  0.8390293  2.0207050  1.4298672
```

We completed this process for many species expected in the region of interest to determine a range of data-generating values for our simulation study and we recommend a similar approach.

Re-create results from Appendix A

The function `compare_3m` applied to `dat` (`allOregonData` processed by `format_query`) will re-create our analysis of the Oregon bat acoustic data. The argument `spp_in` allows the user to choose the species they wish to display results for - default will show all species detected by the classification software.

```
# fit the three models and plot the results
bat_compare <- compare_3m(data = dat, discard = gc_drop,
                          fontsize = 16)
```

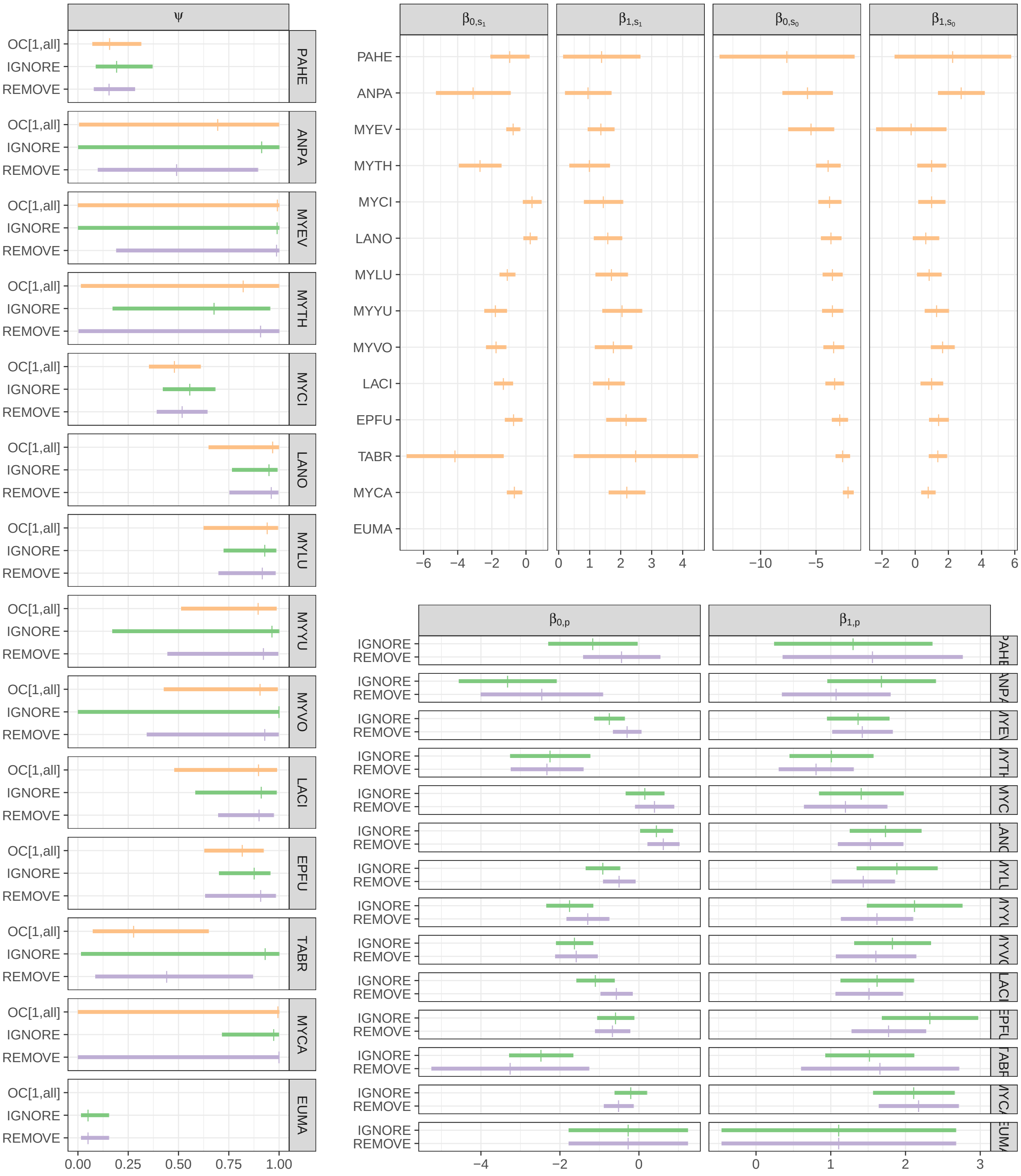


```
# view estimates and CI endpoints
bat_compare
```

Do not be alarmed by the following warning messages:

- `In nlm(p = theta, f = oc_lhood_covdf, df = dat_df, hessian = T) :`

`NA/Inf replaced by maximum positive value` (see *Step1: obtain a range of reasonable data-generating values*).

- `1 sites have been discarded because of missing data`: This is a warning from fitting the standard occupancy model using `occu`. It indicates that there was one site with no data, so it was discarded. This can be ignored.
- `In data.row.names(row.names, rowsi, i) : some row.names duplicated: 6,7,8,9,10,11,12,13,14,15 row.names NOT used`: This warning results from combining results from each species into one data frame for plotting and printing out results. A new variable containing this information is incorporated into the data frame, and this warning can be ignored.
- **Take heed to other warning messages and investigate them!**

Table 1: Estimates and 95% confidence intervals for each of the five species investigated for the three approaches. *Note the order does not match the order in the figure.*

spp	model	pars	est	minus_2SE	plus_2SE
ANPA	REMOVE	psi(Int)	0.4909	0.09895	0.8944
ANPA	REMOVE	p(Int)	-2.46	-4.006	-0.9145
ANPA	REMOVE	p(s1)	1.072	0.3471	1.796
ANPA	IGNORE	psi(Int)	0.9137	0.001024	1
ANPA	IGNORE	p(Int)	-3.324	-4.559	-2.088
ANPA	IGNORE	p(s1)	1.679	0.9537	2.403
ANPA	OC[1,all]	psi	0.6953	0.006131	0.9988
ANPA	OC[1,all]	int_s1	-3.091	-5.271	-0.9116
ANPA	OC[1,all]	int_s0	-5.769	-8.028	-3.511
ANPA	OC[1,all]	slope_s1	0.9496	0.2059	1.693
ANPA	OC[1,all]	slope_s0	2.773	1.373	4.172
EPFU	REMOVE	psi(Int)	0.9087	0.6322	0.9829
EPFU	REMOVE	p(Int)	-0.6696	-1.111	-0.228
EPFU	REMOVE	p(s1)	1.775	1.279	2.272
EPFU	IGNORE	psi(Int)	0.8765	0.7013	0.9555
EPFU	IGNORE	p(Int)	-0.5897	-1.056	-0.1237
EPFU	IGNORE	p(s1)	2.326	1.684	2.968
EPFU	OC[1,all]	psi	0.8176	0.6284	0.9223
EPFU	OC[1,all]	int_s1	-0.7275	-1.235	-0.2198

spp	model	pars	est	minus_2SE	plus_2SE
EPFU	OC[1,all]	int_s0	-2.858	-3.572	-2.144
EPFU	OC[1,all]	slope_s1	2.18	1.534	2.826
EPFU	OC[1,all]	slope_s0	1.42	0.8335	2.007
EUMA	REMOVE	psi(Int)	0.05033	0.01528	0.1533
EUMA	REMOVE	p(Int)	-0.2721	-1.778	1.234
EUMA	REMOVE	p(s1)	1.106	-0.4606	2.673
EUMA	IGNORE	psi(Int)	0.05033	0.01528	0.1533
EUMA	IGNORE	p(Int)	-0.2721	-1.778	1.234
EUMA	IGNORE	p(s1)	1.106	-0.4606	2.673
LACI	REMOVE	psi(Int)	0.9009	0.6971	0.9729
LACI	REMOVE	p(Int)	-0.5693	-0.9725	-0.1662
LACI	REMOVE	p(s1)	1.514	1.063	1.964
LACI	IGNORE	psi(Int)	0.9119	0.5834	0.9871
LACI	IGNORE	p(Int)	-1.101	-1.582	-0.6207
LACI	IGNORE	p(s1)	1.621	1.131	2.111
LACI	OC[1,all]	psi	0.8984	0.4791	0.9884
LACI	OC[1,all]	int_s1	-1.324	-1.872	-0.7773
LACI	OC[1,all]	int_s0	-3.321	-4.149	-2.493
LACI	OC[1,all]	slope_s1	1.617	1.11	2.124
LACI	OC[1,all]	slope_s0	0.9947	0.3243	1.665
LANO	REMOVE	psi(Int)	0.9618	0.7532	0.9952
LANO	REMOVE	p(Int)	0.6175	0.2163	1.019
LANO	REMOVE	p(s1)	1.532	1.095	1.968
LANO	IGNORE	psi(Int)	0.9502	0.7659	0.9911
LANO	IGNORE	p(Int)	0.4432	0.03083	0.8555
LANO	IGNORE	p(s1)	1.733	1.255	2.21
LANO	OC[1,all]	psi	0.9686	0.6497	0.9981
LANO	OC[1,all]	int_s1	0.2519	-0.1474	0.6512
LANO	OC[1,all]	int_s0	-3.644	-4.552	-2.736
LANO	OC[1,all]	slope_s1	1.587	1.135	2.04
LANO	OC[1,all]	slope_s0	0.6427	-0.1438	1.429
MYCA	REMOVE	psi(Int)	1	4.159e-51	1
MYCA	REMOVE	p(Int)	-0.5136	-0.8884	-0.1389
MYCA	REMOVE	p(s1)	2.177	1.643	2.71

spp	model	pars	est	minus_2SE	plus_2SE
MYCA	IGNORE	psi(Int)	0.9734	0.7164	0.9981
MYCA	IGNORE	p(Int)	-0.2072	-0.6149	0.2005
MYCA	IGNORE	p(s1)	2.111	1.567	2.655
MYCA	OC[1,all]	psi	0.9948	8.829e-05	1
MYCA	OC[1,all]	int_s1	-0.6765	-1.117	-0.2365
MYCA	OC[1,all]	int_s0	-2.104	-2.573	-1.636
MYCA	OC[1,all]	slope_s1	2.199	1.613	2.785
MYCA	OC[1,all]	slope_s0	0.7883	0.3628	1.214
MYCI	REMOVE	psi(Int)	0.5183	0.3915	0.6429
MYCI	REMOVE	p(Int)	0.3945	-0.097	0.886
MYCI	REMOVE	p(s1)	1.198	0.6419	1.753
MYCI	IGNORE	psi(Int)	0.556	0.4222	0.6821
MYCI	IGNORE	p(Int)	0.1529	-0.3341	0.6399
MYCI	IGNORE	p(s1)	1.408	0.8436	1.973
MYCI	OC[1,all]	psi	0.4801	0.3535	0.6094
MYCI	OC[1,all]	int_s1	0.3583	-0.1829	0.8995
MYCI	OC[1,all]	int_s0	-3.767	-4.789	-2.745
MYCI	OC[1,all]	slope_s1	1.442	0.8136	2.07
MYCI	OC[1,all]	slope_s0	0.9966	0.1884	1.805
MYEV	REMOVE	psi(Int)	0.9879	0.1904	1
MYEV	REMOVE	p(Int)	-0.2996	-0.6563	0.05708
MYEV	REMOVE	p(s1)	1.423	1.02	1.826
MYEV	IGNORE	psi(Int)	0.9905	0.0005175	1
MYEV	IGNORE	p(Int)	-0.7491	-1.134	-0.3641
MYEV	IGNORE	p(s1)	1.366	0.9504	1.781
MYEV	OC[1,all]	psi	0.9917	3.322e-05	1
MYEV	OC[1,all]	int_s1	-0.7514	-1.15	-0.3525
MYEV	OC[1,all]	int_s0	-5.449	-7.512	-3.386
MYEV	OC[1,all]	slope_s1	1.365	0.9373	1.793
MYEV	OC[1,all]	slope_s0	-0.2447	-2.355	1.865
MYLU	REMOVE	psi(Int)	0.9172	0.6986	0.9815
MYLU	REMOVE	p(Int)	-0.5014	-0.9089	-0.09385
MYLU	REMOVE	p(s1)	1.435	1.014	1.856
MYLU	IGNORE	psi(Int)	0.9289	0.7244	0.9848

spp	model	pars	est	minus_2SE	plus_2SE
MYLU	IGNORE	p(Int)	-0.9134	-1.346	-0.4804
MYLU	IGNORE	p(s1)	1.886	1.346	2.427
MYLU	OC[1,all]	psi	0.9416	0.6247	0.9936
MYLU	OC[1,all]	int_s1	-1.093	-1.546	-0.6396
MYLU	OC[1,all]	int_s0	-3.514	-4.399	-2.63
MYLU	OC[1,all]	slope_s1	1.707	1.189	2.225
MYLU	OC[1,all]	slope_s0	0.843	0.1114	1.575
MYTH	REMOVE	psi(Int)	0.9085	0.003146	1
MYTH	REMOVE	p(Int)	-2.327	-3.246	-1.408
MYTH	REMOVE	p(s1)	0.8041	0.3041	1.304
MYTH	IGNORE	psi(Int)	0.6769	0.1724	0.9547
MYTH	IGNORE	p(Int)	-2.249	-3.26	-1.239
MYTH	IGNORE	p(s1)	1.008	0.4487	1.568
MYTH	OC[1,all]	psi	0.8219	0.0148	0.9993
MYTH	OC[1,all]	int_s1	-2.69	-3.929	-1.451
MYTH	OC[1,all]	int_s0	-3.905	-4.995	-2.814
MYTH	OC[1,all]	slope_s1	0.9934	0.3455	1.641
MYTH	OC[1,all]	slope_s0	0.9906	0.1316	1.85
MYVO	REMOVE	psi(Int)	0.9292	0.3423	0.997
MYVO	REMOVE	p(Int)	-1.586	-2.12	-1.052
MYVO	REMOVE	p(s1)	1.604	1.068	2.14
MYVO	IGNORE	psi(Int)	0.9999	3.784e-25	1
MYVO	IGNORE	p(Int)	-1.631	-2.098	-1.165
MYVO	IGNORE	p(s1)	1.825	1.314	2.336
MYVO	OC[1,all]	psi	0.9053	0.4271	0.9919
MYVO	OC[1,all]	int_s1	-1.752	-2.336	-1.167
MYVO	OC[1,all]	int_s0	-3.406	-4.33	-2.483
MYVO	OC[1,all]	slope_s1	1.765	1.167	2.363
MYVO	OC[1,all]	slope_s0	1.656	0.9464	2.365
MYYU	REMOVE	psi(Int)	0.9226	0.445	0.9944
MYYU	REMOVE	p(Int)	-1.295	-1.834	-0.7557
MYYU	REMOVE	p(s1)	1.618	1.137	2.099
MYYU	IGNORE	psi(Int)	0.9648	0.1706	0.9997
MYYU	IGNORE	p(Int)	-1.754	-2.344	-1.164

spp	model	pars	est	minus_2SE	plus_2SE
MYYU	IGNORE	p(s1)	2.121	1.483	2.759
MYYU	OC[1,all]	psi	0.8962	0.5129	0.9861
MYYU	OC[1,all]	int_s1	-1.787	-2.443	-1.131
MYYU	OC[1,all]	int_s0	-3.51	-4.45	-2.57
MYYU	OC[1,all]	slope_s1	2.046	1.408	2.685
MYYU	OC[1,all]	slope_s0	1.298	0.5783	2.019
PAHE	REMOVE	psi(Int)	0.1554	0.07922	0.2823
PAHE	REMOVE	p(Int)	-0.4394	-1.413	0.5345
PAHE	REMOVE	p(s1)	1.559	0.3564	2.763
PAHE	IGNORE	psi(Int)	0.1931	0.08893	0.3698
PAHE	IGNORE	p(Int)	-1.168	-2.294	-0.04131
PAHE	IGNORE	p(s1)	1.3	0.2429	2.357
PAHE	OC[1,all]	psi	0.158	0.07156	0.3135
PAHE	OC[1,all]	int_s1	-0.9453	-2.084	0.1935
PAHE	OC[1,all]	int_s0	-7.62	-13.69	-1.547
PAHE	OC[1,all]	slope_s1	1.385	0.1436	2.625
PAHE	OC[1,all]	slope_s0	2.262	-1.237	5.761
TABR	REMOVE	psi(Int)	0.4417	0.08633	0.8688
TABR	REMOVE	p(Int)	-3.259	-5.253	-1.264
TABR	REMOVE	p(s1)	1.659	0.6036	2.715
TABR	IGNORE	psi(Int)	0.9314	0.01531	0.9999
TABR	IGNORE	p(Int)	-2.478	-3.287	-1.669
TABR	IGNORE	p(s1)	1.52	0.9264	2.113
TABR	OC[1,all]	psi	0.2773	0.07367	0.6493
TABR	OC[1,all]	int_s1	-4.159	-6.992	-1.327
TABR	OC[1,all]	int_s0	-2.598	-3.233	-1.963
TABR	OC[1,all]	slope_s1	2.486	0.4835	4.488
TABR	OC[1,all]	slope_s0	1.365	0.824	1.906

References

Auguie, Baptiste. 2016. *GridExtra: Miscellaneous Functions for "Grid" Graphics.* https://CRAN.R-project.org/package=gridExtra.

Bache, Sefan Milton, and Hadley Wickham. n.d. *Magrittr: A Forward-Pipe Operator for R.* https://CRAN.R-project.org/package=magrittr.

Fiske, Ian, and Richard Chandler. 2011. "Unmarked: An R Package for Fitting Hierarchical Models of Wildlife Occurance and Abundance." *Journal of Statistical Software* 43 (10): 1–23. http://www.jstatsoft.org/v43/i10/.

Meschiari, Stefano. 2015. *Latex2exp: Use Latex Expressions in Plots.* https://CRAN.R-project.org/package=latex2exp.

Wickham, Hadley. 2007. "Reshaping Data with the reshape Package." *Journal of Statistical Software* 21 (12): 1–20. http://www.jstatsoft.org/v21/i12/.

———. 2009. *Ggplot2: Elegant Graphics for Data Analysis.* Springer New York. http://had.co.nz/ggplot2/book.

Wickham, Hadley, and Romain Francois. 2016. *Dplyr: A Grammar of Data Manipulation.* https://CRAN.R-project.org/package=dplyr.

Wickham, Hadley, Peter Danenberg, and Manuel Eugster. 2017. *Roxygen2: In-Line Documentation for R.* https://CRAN.R-project.org/package=roxygen2.
